# Catalytic Dinitrogen Reduction to Ammonia at a Triamidoamine–Titanium Complex

**DOI:** 10.1002/anie.201802576

**Published:** 2018-04-23

**Authors:** Laurence R. Doyle, Ashley J. Wooles, Lucy C. Jenkins, Floriana Tuna, Eric J. L. McInnes, Stephen T. Liddle

**Affiliations:** ^1^ School of Chemistry The University of Manchester Oxford Road Manchester M13 9PL UK; ^2^ School of Chemistry The University of Nottingham University Park Nottingham NG7 2RD UK; ^3^ School of Chemistry and Photon Science Institute The University of Manchester Oxford Road Manchester M13 9PL UK

**Keywords:** ammonia, dinitrogen reduction, nitrogen fixation, phosphonium salts, titanium

## Abstract

Catalytic reduction of N_2_ to NH_3_ by a Ti complex has been achieved, thus now adding an early d‐block metal to the small group of mid‐ and late‐d‐block metals (Mo, Fe, Ru, Os, Co) that catalytically produce NH_3_ by N_2_ reduction and protonolysis under homogeneous, abiological conditions. Reduction of [Ti^IV^(Tren^TMS^)X] (X=Cl, **1A**; I, **1B**; Tren^TMS^=N(CH_2_CH_2_NSiMe_3_)_3_) with KC_8_ affords [Ti^III^(Tren^TMS^)] (**2**). Addition of N_2_ affords [{(Tren^TMS^)Ti^III^}_2_(μ‐η^1^:η^1^‐N_2_)] (**3**); further reduction with KC_8_ gives [{(Tren^TMS^)Ti^IV^}_2_(μ‐η^1^:η^1^:η^2^:η^2^‐N_2_K_2_)] (**4**). Addition of benzo‐15‐crown‐5 ether (B15C5) to **4** affords [{(Tren^TMS^)Ti^IV^}_2_(μ‐η^1^:η^1^‐N_2_)][K(B15C5)_2_]_2_ (**5**). Complexes **3**–**5** treated under N_2_ with KC_8_ and [R_3_PH][I], (the weakest H^+^ source yet used in N_2_ reduction) produce up to 18 equiv of NH_3_ with only trace N_2_H_4_. When only acid is present, N_2_H_4_ is the dominant product, suggesting successive protonation produces [{(Tren^TMS^)Ti^IV^}_2_(μ‐η^1^:η^1^‐N_2_H_4_)][I]_2_, and that extruded N_2_H_4_ reacts further with [R_3_PH][I]/KC_8_ to form NH_3_.

The conversion of dinitrogen, N_2_, into ammonia, NH_3_, is essential for supplying N_2_ in a fixed form into the Earth's biosphere,^1^ and key to providing NH_3_ to chemical industry on a vast scale.^2^ However, the N≡N triple bond, with a bond strength of 944 kJ mol^−1^, is one of the strongest chemical bonds known, and with a high ionisation potential, negative electron affinity, poor nucleophilicity and electrophilicity, large HOMO–LUMO gap, and no permanent dipole, there are major kinetic and thermodynamic barriers to activating N_2_, and thus to fixing it as NH_3_.^3^, ^4^

Nature uses homogeneous nitrogenases based on V, Mo, and Fe to execute multiple single‐electron transfer and protonation steps to convert N_2_ into NH_3_.^5^ In contrast, chemical industry uses N_2_ and H_2_ to produce NH_3_ over heterogeneous catalysts in the Haber–Bosch process.^6^ However, each process is energy‐intensive, reflecting the challenge of N_2_ activation, and so there is interest in studying reactivity at molecular complexes to improve our understanding of these elementary transformations.^7^

After the report that a molybdenum–triamidoamine complex catalytically reduces N_2_ to NH_3_ in the presence of H^+^/e^−^,^8^ a few Mo, Fe, Ru, Os, and Co complexes have been shown to be catalytically competent in H^+^/e^−^ mediated N_2_ reduction cycles,^9^ and stoichiometric reduction and protonation/hydrogenation of N_2_ and nitrides by a variety of metals have been reported.^10^ However, no abiological, early metal complex preceding Group 6 has ever been shown to catalytically convert N_2_ into NH_3_, and even V‐, Cr‐, and W‐triamidoamine complexes^11^ do not facilitate N_2_ reduction/protonolysis to NH_3_ like Mo analogues.^8^ Where Ti is concerned, cleavage of N_2_ by molecular polyhydrides and low valent species and/or stoichiometric protonolysis has been reported.^12^ Recently, a heterogeneous Ti hydride was found to catalytically convert N_2_ and H_2_ into NH_3_, whilst TiO_2_ is known to photolytically convert N_2_ and H_2_O into NH_3_ and O_2_.^13^ These reports hint that Ti could hold significant promise in this arena. This is appealing because Ti is the ninth most abundant element in the Earth's crust, and second only to Fe for metals that can fix N_2_.

Herein, we report the first abiological, homogeneous Ti complex that is competent for catalytic reduction of N_2_ to NH_3_. We find that N_2_ binding and partial activation occurs at Ti^III^ supported by one of the simplest triamidoamine ligands, priming the N_2_ for cooperative reduction by KC_8_. Protonation to give NH_3_ in a catalytic cycle is facilitated by phosphonium salts that are the weakest proton source used in any catalytic system to date.

Under argon, reduction of yellow [Ti^IV^(Tren^TMS^)X] (X=Cl, **1 A**;^14^ I, **1 B**; Tren^TMS^=N(CH_2_CH_2_NSiMe_3_)_3_) with KC_8_ yields green [Ti^III^(Tren^TMS^)] (**2**).^15^ Three broad ^1^H NMR resonances are observed (C_6_D_6_, 3.6, 1.0, and −22.3 ppm), whilst no ^13^C or ^29^Si NMR resonances could be detected, and an EPR spectrum of **2** in frozen toluene‐pentane exhibits *g=*1.984, 1.943, and 1.891. This is consistent with the presence of Ti^III^, unfortunately **2** has resisted exhaustive attempts to isolate it. However, slow cooling of an N_2_‐saturated pentane solution of **2** yields red crystals of [{(Tren^TMS^)Ti^III^}_2_(μ‐η^1^:η^1^‐N_2_)] (**3**), Scheme 1.^15^ In the solid state **3** has an effective magnetic moment of 2.50 μ_B_ at 298 K, which is close to the spin‐only value for two *s*=1/2 species (*μ*_eff_=2.5 β_e_ for *g=*2.0). The near temperature‐independence of *μ*_eff_ indicates that any Ti⋅⋅⋅Ti interaction is very weak.^15^ EPR spectra of solid **3**, give apparently axial *g*_⊥_=1.989 and *g*_∥_=1.940 at room temperature; however on cooling these diverge to rhombic *g=*1.993, 1.951, and 1.902 and are temperature‐independent below 100 K, suggesting that molecular motion at higher temperatures gives higher effective symmetry. The magnetism and EPR spectra of **3** thus suggest the presence of Ti^III^ ions, and the structural and spectroscopic data (Table 1) are consistent with modest activation of the N≡N triple bond.^16^

**Scheme 1:**
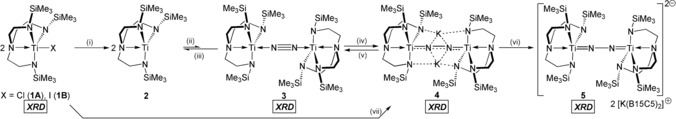
Synthesis of **2**–**5** from **1A/B**. Reagents and conditions: i) Ar, 2 KC_8_, −2 KX, −2 C_8_; ii) N_2_, cool; iii) −N_2_, warm; iv) 2 KC_8_; v) I_2_, −2 KI; vi) 4 B15C5; vii) N_2_, 4 KC_8_, −2 KX, −2 C_8_.

**Table 1:** Key crystallographic bond lengths and Raman spectroscopic data for **3**–**5** and N_2_H_*x*_ benchmarks (*x=*0, 2, 4).^15^, ^24^

Cmpd	*ν*(N_2_) [cm^−1^] exp(^14^N/^15^N); calc	*d*(N−N) [Å]	*d*(Ti−NN) [Å]	∡(Ti−N−N) [°]
N_2_	2330	1.098(1)	–	–
N_2_H_2_	1583,1529	1.25	–	–
N_2_H_4_	1076	1.45	–	–
**3**	1701/1644;1724	1.121(6)	2.022(3)	180
**4**	1201/1164;1247	1.315(3)	1.814(2) 1.810(2)	166.6(2) 172.9(2)
**5**	1246/1203;1307	1.461(7)	1.712(4)	178.8(5)

The formulation of **3** is corroborated by DFT calculations;^15^ all attempts to model a Ti^IV^/Ti^IV^/N_2_^2−^ combination were intractable or produced the Ti^III^/Ti^III^/N_2_ formulation. Computed Ti−NN and N−N Mayer bond orders of 0.56 and 2.38 are consistent with a weakly bound and activated N_2_. This is also supported by computed Ti spin densities/charges of −0.55/0.99 and a total net spin density/charge of −0.9/‐0.56 on the N_2_‐unit. The two SOMOs are orthogonal Ti→N_2_ π* back‐bonding interactions.

The ^1^H NMR spectrum of **3** (C_6_D_6_) reveals identical resonances to those of **2**, consistent with N_2_ dissociation in solution. Complex **2** is similar to [Ti(Tren^DMBS^)] [Tren^DMBS^=N(CH_2_CH_2_NSiMe_2_Bu^t^)_3_],^17^ and their optical spectra exhibit broad absorptions at about 620 and about 640 nm, respectively,^15^ which is responsible for their green colours. In line with this, [Ti(Tren^TMS^)] exhibits computed HOMO to LUMO+3/+4 (d–d) energy separations of 655/657 nm.

Addition of two or four equivalents of KC_8_ to **3** or **1A**, respectively, under N_2_ affords red–brown [{(Tren^TMS^)Ti^IV^}_2_(μ‐η^1^:η^1^:η^2^:η^2^‐N_2_K_2_)] (**4**; Scheme 1).^15^ The structure of **4** (Table 1) shows the N_2_ ligand is bound in a near‐linear manner. The N−N bond distance is extended compared to **3** and free N_2_ and the Ti−NN bond distances are short, inferring some Ti–imido character. These data along with Raman spectroscopy suggest strong activation of N_2_. Multinuclear NMR spectra of C_6_D_6_ solutions of **4** and **4**‐^**15**^ **N_2_** are consistent with a *C*_3_‐symmetric Ti^IV^/Ti^IV^/N_2_^4−^ formulation.

Only a closed‐shell formulation for **4** gave a converged DFT calculation,^15^ where the Ti ions and N_2_ unit carry computed charges of 0.52 (av.) and −1.1 (total), respectively; this implies a covalent bonding picture for the Ti−(μ‐N_2_)−Ti unit that is supported by Ti−NN and N−N Mayer bond orders of 1.25 and 1.44, respectively. For comparison, the Ti−N_amide_ and Ti−N_amine_ Mayer bond orders are about 0.7 and 0.25, respectively. The HOMO and HOMO−1 are two orthogonal, doubly occupied Ti→N_2_ π* back‐bonding interactions. The K^I^ ions clearly play a stabilising role, but this is electrostatic, with K−N Mayer bond orders of <0.05.

Addition of 4 equivalents of benzo‐15‐crown‐5 (B15C5; Scheme 1) gives red [{(Tren^TMS^)Ti^IV^}_2_(μ‐η^1^:η^1^‐N_2_)][K(B15C5)_2_]_2_ (**5**).^15^ The solid‐state molecular structure of **5** (Table 1) reveals that the Ti−N−N−Ti axis is closer to linearity than in **4**. The N−N and Ti−NN bond distances in **5** are longer and shorter, respectively, suggesting strong N_2_ activation and significant Ti–imido character. However, for **5** the *ν*(N_2_) Raman stretch, a better indicator of N_2_ activation than bond distances, suggests reduced N_2_ activation compared to **4**, Table 1. UV/Vis spectra of **4** and **5** are essentially identical in THF, but different in benzene, suggesting that the K^I^ ions in **4** are labile in polar donor solvent, but remain coordinated in non‐polar solvents.

Only a closed‐shell formulation for the dianion part of **5** gave a converged calculation. The Ti−NN and N−N Mayer bond orders in **5** are 1.33 and 1.62, which are larger than the corresponding data for **4**; the latter is in‐line with the Raman data, whilst the former suggests that more Ti–imido character results from removal of K^I^ ions. The fact that **5** contains a charge‐rich dianion is reflected by Ti−N_amide_ and Ti−N_amine_ Mayer bond orders that are lower than in **4** at about 0.6 and 0.16, respectively. This is consistent with computed Ti charges of 0.99 in **5** that are higher than those in **4**, but the N_2_ unit is less charged at −0.86 overall.

Having established N_2_ reduction, attention turned to fixation (Table 2). Treatment of **4** (10 mm, pentane) with ethereal 1 m HCl (10 equiv) yielded 0.88 N_2_H_4_ and 0.13 NH_3_ equiv (entry 1). The near‐stoichiometric yield of N_2_H_4_ confirms **4** as a hydrazido complex. In a control, when **1A** is identically quenched only 0.04 NH_3_ equiv were detected. This suggests that the small amount of NH_3_ produced is the result of minor degradation of Tren^TMS^ under the action of very strong acid.

**Table 2:** Catalytic acidification experiments for the reaction of **4** with acid and reductant under N_2_ to produce NH_3_ and N_2_H_4_.^[a]^

Entry^[b]^	Solvent	Acid	Reductant	Acid [eq.]	Reductant [eq.]	NH_3_ [eq.]	N_2_H_4_ [eq.]	Fixed‐N^[h]^ [eq.]	Efficiency^[i]^ [%]
1	pentane	1 m HCl	–	10	–	0.13	0.88	1.89	–
2	Et_2_O	[Cy_3_PH][I]	–	10	–	0.05	0.52	1.09	–
3	Et_2_O	[Cy_3_PH][I]	KC_8_	120	120	6.41	0.15	6.71	17
4	Et_2_O	[Cy_3_PH][I]	KC_8_	300	300	11.91	0.06	12.03	12
5	Et_2_O	[Cy_3_PH][I]	KC_8_	400	300	10.81	0.10	11.01	11
6	Et_2_O	[Cy_3_PH][I]	KC_8_	600	600	17.77/17.4^[g]^	0.03	17.83	9
7^[c,d]^	Et_2_O	[Cy_3_PH][I]	KC_8_	600	600	17.70/17.6^[g]^	0.08	17.86	9
8^[c,e]^	Et_2_O	[Cy_3_PH][I]	KC_8_	300	300	1.53	0	1.53	–
9	pentane	[Cy_3_PH][I]	KC_8_	300	300	5.82	0.29	6.40	6
10	toluene	[Cy_3_PH][I]	KC_8_	300	300	3.89	0.21	4.31	4
11	THF	[Cy_3_PH][I]	KC_8_	300	300	8.95	0	8.95	9
12	Et_2_O	[Cy_3_PH][I]	K(Nap)(THF)	300	300	0.36	0.07	0.50	0
13	Et_2_O	[Cy_3_PH][Cl]	KC_8_	300	300	2.45	0.05	2.55	3
14	Et_2_O	[Cy_3_PH][BAr^F^_4_]	KC_8_	300	300	4.77	0	4.77	5
15	Et_2_O	[^n^Bu_3_PH][I]	KC_8_	300	300	11.73	0.09	11.91	12
16	Et_2_O	[^t^Bu_3_PH][I]	KC_8_	300	300	7.37	0.30	7.97	8
17^[f]^	Et_2_O	[Cy_3_PH][I]	KC_8_	25	25	1.32	0.34	2.00	–

[a] Diazene (N_2_H_2_) was not analysed owing to its expected instability under these reaction conditions; however, complete disproportionation of N_2_H_2_ to N_2_H_4_ and N_2_ can only be expected to produce a maximum N_2_H_4_ yield of 50 %. [b] All experiments were performed under N_2_ (unless otherwise noted) at −78 °C (2 h), followed by gradual warming to 25 °C and additional stirring for 15 h. [c] **4**‐^**15**^ **N_2_**. [d] Under ^15^N_2_. [e] Under Ar. [f] [N_2_H_5_][I]. [g] Yield calculated from ^1^H NMR. [h] Fixed‐N (eq.)=[NH_3_ (eq.)]+2 [N_2_H_4_ (eq.)]. [i] Efficiency=100 %[Red. (eq.)]/{3[NH_3_ (eq.)]+4 [N_2_H_4_ (eq.)]}.

To achieve catalytic turnover of N_2_, it was concluded that a milder acid than ethereal HCl (p*K*_a_ (Et_2_O⋅H)^+^=−3.59) would be required. Also, the synthesis of **4** requires strong K‐based reductants, which react rapidly with strong, soluble acids. Confirming this, using HCl/KC_8_ (30:30:**4**) resulted in a sub‐stoichiometric yield of N_2_H_4_ and NH_3_ (0.13 and 0.36 equiv, respectively). It was anticipated that a weaker acid could be effective for the protonation of activated N_2_ whilst minimising deleterious side‐reactions. However, commonly used *N*‐based acids, such as lutidinium [2,6‐(CH_3_)_2_C_5_H_3_NH]^+^ and arylammonium/alkylammonium salts were found to be susceptible to reduction by KC_8_, and in some controls led to NH_3_ formation; as such, we anticipated false‐positive results. Thus, more stable, non‐*N*‐based acids were sought.

Trialkylphosphonium salts, [R_3_PH][X], were examined because of their mild acidity (p*K*_a_≈8–12) and tuneable nature. Initial experiments were conducted with [Cy_3_PH][I] (p*K*_a_=9.7). Addition to **4** (10:1 ratio) in Et_2_O produced 0.5 N_2_H_4_ and 0.05 NH_3_ equiv (entry 2). This is a lower yield of N_2_H_4_ compared to using HCl, but [Cy_3_PH][I] is a weaker, less soluble acid so it was expected to be less reactive. However, with excess [Cy_3_PH][I]/KC_8_, **4** catalyses the production of up to 18 equiv of NH_3_ per **4** (entries 3–11). Using **4**‐^**15**^ **N_2_** under ^15^N_2_ confirmed the incorporation of ^15^N_2_ into the ^15^NH_3_/^15^NH_4_Cl product.

A sub‐stoichiometric yield was obtained using [K_2_(C_10_H_8_)_2_(THF)] as the reductant (entry 12), which is most likely due to its solubility in Et_2_O and thus greater propensity to react with H^+^ in situ, consuming acid and reductant for H_2_ production. Comparative runs in ethers (Et_2_O and THF), in which intermediates may be solvated/stabilised, gave higher yields than those in toluene or pentane (entries 4 and 9–11). The acid anion was also varied: [Cy_3_PH][Cl] (Cl^−^, higher coordinating ability) or [Cy_3_PH][BAr^F^_4_] (BAr^F^_4_^−^=[{3,5‐(CF_3_)_2_C_6_H_3_}_4_B]^−^, non‐coordinating; entries 4, 13, and 14). For both, this resulted in lower yields of NH_3_, but supposed variation of a single parameter will simultaneously affect several properties that all influence catalytic turnover, preventing meaningful correlation. Similar catalytic turnovers were obtained using [Bu^n^_3_PH][I] and [Bu^t^_3_PH][I] (p*K*_a_=8.4 and 11.4, respectively; entries 15 and 16), or using a 4:3 ratio of [Cy_3_PH][I] to KC_8_ (entry 5), which accounts for an additional equiv of acid being consumed through protonation of NH_3_.

With KC_8_, the major product is NH_3_, with little N_2_H_4_ (<0.15 per **4**) detected. That **4** does not react further with KC_8_ in Et_2_O implicates an initial protonation step rather than further reduction of **4** to form a Ti^IV^–nitride species, which could be responsible for NH_3_ production. A further control demonstrated that [N_2_H_5_][I] is converted into NH_3_ in the absence of **4**/**4**‐^**15**^ **N_2_** (entry 17), and with entry 1, this suggests that the N_2_H_4_ to NH_3_ reduction/protonation may occur after dissociation from the active species. We cannot rule out the presence of transient Ti^IV^–nitrides,^18^ but these control experiments suggest that successive protonation may produce “[{(Tren^TMS^)Ti^IV^}_2_(μ‐η^1^:η^1^‐N_2_H_4_)][I]_2_”, with subsequent extrusion of N_2_H_4_, that reacts with [Cy_3_PH][I]/KC_8_ to form NH_3_; concomitant formation of **1B** closes the catalytic cycle.

Under conditions given in entry 4, **5** is catalytically competent producing 10 equiv of NH_3_. Complex **3** produces 6 equiv of NH_3_ under such conditions, showing catalytic competence, but on dissolution a significant proportion of **3** converts into **2** (Scheme 1), retarding reactivity. In isolation, **3** reacts with acid to produce 0.03 and 0.1 equiv of NH_3_ and N_2_H_4_, respectively, underscoring the importance of KC_8_ activation of the coordinated N_2_ in **3**.

The reactivity of **5** is significant, because it is a clear‐cut end‐on:end‐on bridging N_2_ complex. To date, almost all N_2_‐fixing catalysts contain end‐on terminal N_2_,^7^ whereas those with end‐on:end‐on bridging N_2_ have been proposed to dissociate to form end‐on terminal complexes, or undergo N_2_ cleavage to generate nitrides.^7^, 9b,9d, 18, 19, 20 Side‐on:side‐on bridging, which usually results in strong N_2_ activation, has been reported for stoichiometric N_2_ activation only. Conversion of end‐on:end‐on bridging into side‐on:side‐on bridging in **5** seems unlikely on steric grounds, so the reactivity of **5** suggests that consideration might be given to recognising end‐on:end‐on bridging as a catalytically competent N_2_ coordination mode. If this is the case, it would provide an unusual symmetrical functionalisation of N_2_ to N_2_H_4_ (in the absence of reductant to cleave the N−N bond) compared to the traditional Chatt‐type cycle,^21^ since only one molecular Fe complex is known to be selective for catalytic reduction of N_2_ to N_2_H_4_ rather than NH_3_.^22^

To conclude, we have reported the first abiological early base metal complex that is competent for the homogeneous catalytic reduction of N_2_ to NH_3_ under ambient conditions, and this chemistry is supported by the simple Tren^TMS^ ligand. The proton sources in this catalysis, [R_3_PH][I], are the weakest yet used in N_2_ reduction to NH_3_, underscoring the importance of balancing acid sources to strong reducing agents. We propose a plausible mechanism, where Ti^IV^ is reduced to Ti^III^, which binds and weakly activates N_2_, priming it for cooperative K‐mediated reduction10e, 23 to a strongly activated state, which can then be protonated. Transient nitrides cannot be ruled out at this point, but control experiments suggest that N_2_H_4_ is formed and then converted into NH_3_ in the presence of acid and reductant. The catalytic activity of one of the Ti complexes suggests that the end‐on:end‐on bridging mode of N_2_ should possibly no longer be discounted as a catalytically active coordination mode. These results add Ti to the small number of previously exclusively mid‐ and late‐d‐block metal ions (Mo, Fe, Ru, Os, Co) that can execute catalytic reduction of N_2_ to NH_3_.^24^

## Conflict of interest

The authors declare no conflict of interest.

## Supporting information

As a service to our authors and readers, this journal provides supporting information supplied by the authors. Such materials are peer reviewed and may be re‐organized for online delivery, but are not copy‐edited or typeset. Technical support issues arising from supporting information (other than missing files) should be addressed to the authors.

Supplementary
