# Supplementary material for: Catalytic Dinitrogen Reduction to Ammonia at a Triamidoamine–Titanium Complex
Source: Angew Chem Int Ed Engl. 2018 Apr 23;57(21):6314–8. doi: 10.1002/anie.201802576 (PMC6003280; doi:10.1002/anie.201802576)
Supplement: Supplementary file 1 — Supplementary [file ANIE-57-6314-s001.pdf]

Angewandte Chemie
Eine Zeitschrift der Gesellschaft Deutscher Chemiker

Supporting Information

Catalytic Dinitrogen Reduction to Ammonia at a Triamidoamine–Titanium Complex

*Laurence R. Doyle, Ashley J. Wooles, Lucy C. Jenkins, Floriana Tuna, Eric J. L. McInnes, and Stephen T. Liddle**

anie_201802576_sm_miscellaneous_information.pdf

Experimental details

General considerations

Unless stated otherwise, all manipulations were carried out under an inert N_2 or Ar atmosphere using either a dual-bank vacuum and N_2/Ar gas manifold, or a MBraun UniLab glovebox (N_2). Solvents were dried using a Innovative Technology Pure Solv™ SPS-400, degassed under dynamic vacuum, and stored over either a K mirror (pentane, hexane, toluene, Et_2O) or activated 4 Å molecular sieves (THF). C_6D_6 was distilled from K, freeze-pump-thaw degassed, and stored under N_2; DMSO-d_6 was dried over five rounds of activated 3 Å molecular sieves, freeze-pump-thaw degassed, and stored under N_2.

PCy_3, P^nBu_3, P^tBu_3, HCl (1 M in Et_2O), NH_4Cl, $N_2H_4{\cdot}2HCl$, NH_3 (0.4 M in THF), N_2H_4 (1 M in THF), TMSCl (TMS = $SiMe_3$), TMSI, PhOH, NaOH, KOH, NaOCl, and $Na_2[Fe(CN)_5NO]$ were purchased from commercial suppliers and used as received. Benzo-15-crown-5 (B15C5) was dried under dynamic vacuum at 10^{-3} mbar for 5 h prior to use. Ti(Tren^{TMS})(Cl) [**1A**; Tren^{TMS} = $\{N(CH_2CH_2NSiMe_3)_3\}^{3-}$],[1] Ti(Tren^{DMBS})(Cl) [Tren^{DMBS} = $\{N(CH_2CH_2NSiMe_2{}^tBu)_3\}^{3-}$],[2] KC_8,[3] potassium napthalenide $[K_2(C_{10}H_8)_2(THF)]$,[4] and $[H(OEt_2)_2][BAr^F_4]$ ($BAr^F_4{}^-$ = $[B\{3,5\text{-}(CF_3)_2C_6H_3\}_4]^-$)[5] were prepared according to previously reported methods. HI (1 M in Et_2O) was prepared *in situ* by combining TMSI and ^tBuOH (1:1) in Et_2O. $[N_2H_5]I$ was prepared *via* slow addition of HI (1 M in Et_2O) to N_2H_4 (1 M in THF); the mixture was cooled to –78 °C and the white crystalline $[N_2H_5]I$ was collected by filtration, washed with further Et_2O and then dried *in vacuo*. Phosphonium salts (R_3PHX; R = Cy, ^nBu, ^tBu; X = Cl, I, BAr^F_4) were prepared *via* slow addition of HX {HCl, HI, or $[H(OEt_2)_2][BAr^F_4]$; all 1 M Et_2O solutions} to a Et_2O solution of the phosphine (pre-cooled to –78 °C); the precipitated white phosphonium salt was collected by filtration, washed with further Et_2O, and dried *in vacuo*.

Physical methods

NMR spectra were recorded on Bruker 400/500 spectrometers operating at 400.1/500.1 MHz (^{1}H), 100.6/125.8 MHz (^{13}C), 40.6/50.7 MHz (^{15}N), 162.0/202.5 MHz (^{31}P), and 79.5/99.4 (^{29}Si) MHz. ^{1}H and ^{13}C chemical shifts were referenced (δ = 0) internally to the residual solvent peaks of the deuterated solvent employed and quoted relative to $SiMe_4$. ^{15}N, ^{29}Si, and ^{31}P chemical shifts were referenced (δ = 0) externally to $MeNO_2$, $SiMe_4$, and 85% H_3PO_4 (aq), respectively. FTIR spectra were recorded using a Bruker Alpha spectrometer using either a Specac Omni-Cell™ or a Bruker attenuated total reflection (ATR) accessory for solution and powder measurements, respectively. Raman spectra were recorded using a Horiba XploRA™PLUS Raman microscope. Samples were measured as powders using a 633 nm laser at 1% intensity. UV-vis spectra were recorded on a Perkin Elmer Lambda 750 spectrometer. Samples were prepared under N_2 in a sealed 1 mm path length cuvette; background subtractions were applied for each sample. Variable temperature (5-300 K) EPR spectra were measured at X-band (ca 9.4 GHz) on a Bruker EMX 300 spectrometer. Powder measurements were performed on a polycrystalline sample, which was ground to a fine powder and flame sealed under vacuum within a 1 mm i.d. quartz EPR tube. Frozen glass measurements were performed on frozen toluene/pentane (95:5) solutions, which were flame sealed under vacuum within a 2.8 mm i.d. quartz EPR tube. Static variable temperature magnetic moment data were recorded in an applied dc field of 1 kG on a Quantum Design MPMS XL7 superconducting quantum interference device (SQUID) magnetometer. The sample was immobilised in an eicosane matrix to prevent sample reorientation during measurements. Diamagnetic corrections were applied using tabulated Pascal constants, and data were also corrected for the effect of the blank sample holder (flame sealed Wilmad NMR tube) and eicosane matrix. Elemental analyses (CHN) were performed by Mr S. Boyer of the London Metropolitan University.

Synthesis of [Ti^{III}($Tren^{TMS}$)] (2), in situ, and [{($Tren^{TMS}$)Ti^{III}}$_2$(μ-η^1:η^1-N_2)] (3)

Method 1: Manipulations were carried out under a N_2 atmosphere. A pentane solution of [{($Tren^{TMS}$)Ti^{IV}}$_2$(μ-η^1:η^1:η^2:η^2-N_2K_2)] (**4**, 921.5 mg, 1 mmol in 20 mL) was cooled to –78 °C in a dry ice/acetone bath. Before **4** could crystallise from the solution, I_2 was added dropwise as a dilute

pentane solution (254 mg, 1 mmol in 20 mL). A brown/green solution formed at –78 °C which turned deep emerald green upon warming to room temperature. The solution was left stirring overnight and then filtered to remove KI. The resulting solution of **2** was concentrated *in vacuo* then cooled to –30 °C under a N_2 atmosphere for 15 h, yielding **3** as a green powder, which was washed with additional cold pentane (–78 °C, 3 x 10 mL) and dried *in vacuo*. Yield: 540 mg, 64%. Anal. calcd. for $C_{30}H_{78}N_{10}Si_6Ti_2$: C 42.73; H 9.32; N 16.61%; Found: C 42.92; H 9.54; N 16.75%. ^{1}H NMR (C_6D_6, 298 K): δ (ppm): 3.57 (br s, 54H, CH_3), 1.03 (br s, 12H, CH_2), –22.37 (br s, 12H, CH_2). Raman [powder; ν(N_2)]: ν/cm^{-1}: 1701 (s). ATR-FTIR (powder): ν/cm^{-1}: 2947 (s), 2882 (s), 2846 (s), 1460 (w), 1448 (w), 1401 (w), 1360 (w), 1327 (s), 1259 (w), 1244 (s), 1150 (w), 1116 (w), 1044 (s), 927 (s), 898 (s), 823 (s), 783 (s), 740 (s), 688 (s), 670 (s), 622 (w), 567 (s), 524 (s), 474 (s), 438 (w), 407 (s). UV/Vis: λ_{max}/nm (ε/M^{-1}cm^{-1}): toluene, 614 (287), 1,241 (82). *Method 2:* Inside a N_2 glovebox, Ti^{IV}(Tren^{TMS})(Cl) (**1A**, 443.1 mg, 1 mmol) and KC_8 (148.7 mg, 1.1 mmol) were weighed into a Schlenk, which was then transferred to a dual-bank vacuum and Ar gas manifold. After the Schlenk was degassed of N_2 and subsequently cooled in a LN_2/pentane bath (–131 °C), near-frozen Ar-saturated pentane was added to the frozen solids under a flow of Ar. The mixture was then submerged in a dry-ice/acetone bath (–78 °C), and left to warm to room temperature with stirring over a period of 2 hours, followed by a further 15 h stirring at room temperature. The resulting solution was filtered, then concentrated *in vacuo* to yield **2** as a green oil. Exposure of **2** to N_2 (1 bar) yielded **3** as a green powder, which was washed with additional cold pentane (–78 °C, 3 x 10 mL) and dried *in vacuo*. Yield: 196 mg, 47 %.

$^{15}N_2$ enrichment of 3

Complex **3** (80 mg, 0.095 mmol) was weighed into an ampoule and then dissolved in pentane (5 mL) to form a green solution. After concentration *in vacuo* to ca 0.2 mL, the saturated solution/slurry was frozen in a LN_2 bath (–196 °C) and the ampoule was sealed under a static vacuum. $^{15}N_2$ (1 bar, 780 eq.) was admitted to the headspace of the ampoule using a Toepler pump. The mixture was then warmed to room temperature, stirred for 30 min, then cooled to –78 °C and stirred for a further 30 min. The remaining volatiles were subsequently removed *in vacuo* yielding a green solid/oil, which

was then transferred into a N_2 glovebox. After exposure to N_2, this converted to a green powder, which was spectroscopically identical to **3** except for an additional Raman band at 1644 cm^{-1}, corresponding to the isotopically shifted $\nu(^{15}N^{15}N)$ stretch $\{\nu(^{14}N^{14}N) \times [\mu(^{14}N^{14}N)/\mu(^{15}N^{15}N)]^{0.5} = 1643\ cm^{-1}\}$.

Synthesis of $[\{(Tren^{TMS})Ti^{IV}\}_2(\mu\text{-}\eta^1:\eta^1:\eta^2:\eta^2\text{-}N_2K_2)]$ (4)

Inside a N_2 glovebox, **1A** (3 g, 6.77 mmol) and KC_8 (2.746 g, 20.31 mmol) were weighed into a Schlenk, which was then transferred to a dual-bank vacuum and N_2 gas manifold. After submerging the Schlenk in a dry-ice/acetone bath (–78 °C), pre-cooled hexane (50 ml, –78 °C) was added *via* cannula transfer under a flow of N_2, and the mixture was subsequently left to warm to room temperature with stirring over a period of 2 hours. After a further 3 days of stirring at room temperature, filtration through Celite® afforded a reddish brown solution. The hexane solvent was removed *in vacuo* until the solution was saturated, whereupon cooling to –30 °C for 15 h yielded reddish brown crystals of **2**; these were collected by filtration, washed with pre-cooled hexane (3 x 10 mL, –78 °C), and then dried *in vacuo*, yielding **2** as a microcrystalline brown powder. Yield: 2.375 g, 76%. Anal. calcd. for $C_{30}H_{78}K_2N_{10}Si_6Ti_2$: C 39.10; H 8.53; N 15.20%; Found: C 38.96; H 8.43; N 15.06%. ^1H NMR (C_6D_6, 298 K): δ (ppm): 3.09 (t, 12H, CH_2), 2.40 (t, 12H, CH_2), 0.32 (s, 54H, CH_3). $^{13}C\{^1H\}$ NMR (C_6D_6, 298 K): δ (ppm): 62.59 (s, CH_2), 47.98 (s, CH_2), 4.06 (s, CH_3). $^{29}Si\{^1H\}$ NMR (C_6D_6, 298 K): δ (ppm): –4.33. Raman (powder): ν/cm^{-1}: 99 (s), 185 (w), 210 (w), 252 (w), 312 (w), 345 (w), 378 (s), 425 (w), 453 (w), 507 (w), 610 (w), 730 (w), 1201 (vs), 2824 (w), 2848 (w), 2893 (w), 2944 (w). ATR-FTIR (powder): ν/cm^{-1}: 2939 (s), 2891 (w), 2844 (s), 2811 (s), 1462 (w), 1444 (w), 1341 (w), 1237 (s), 1152 (w), 1058 (s), 1046 (s), 1026 (w), 1008 (w), 952 (s), 941 (s), 925 (s), 899 (s), 823 (s), 759 (vs), 733 (s), 678 (s), 656 (s), 611 (w), 573 (w), 559 (s), 541 (w), 466 (w), 455 (w), 433 (s). UV/Vis: λ_{max}/nm ($\varepsilon/M^{-1}cm^{-1}$): pentane, 319 (30,460); C_6H_6, 319 (32,012); Et_2O, 315 (30,320); THF, 312 (28,980), 394 (6,440).

Synthesis of [{(Tren^{TMS})Ti^{IV}}$_2$(μ-η^1:η^1:η^2:η^2-^{15}N$_2$K$_2$)] (4-^{15}N$_2$)

Inside a N_2 glovebox, **1A** (443.1 mg, 1 mmol) and KC_8 (405.6 mg, 3 mmol) were weighed into an ampoule fitted with a modified J. Young valve/ Quickfit® side arm, which was then transferred to a dual-bank vacuum and Ar gas manifold. After the ampoule was degassed of N_2 and subsequently cooled in a LN_2/pentane bath (–131 °C), near-frozen Ar-saturated hexane (ca –90 °C) was added to the frozen solids under a flow of Ar. After freezing the mixture and evacuating the headspace, the ampoule was connected to a Toepler line and $^{15}N_2$ gas (1 bar, 2.4 eq.) was added to the headspace. The mixture was thawed by submerging the ampoule in a dry-ice/acetone bath (–78 °C), and then left to warm to room temperature with stirring over a period of 2 hours, followed by a further 3 days stirring at room temperature. **4-^{15}N$_2$** was subsequently isolated using the same procedure as for **4**. Yield: 302 mg, 66%. ^{15}N NMR (C_6D_6, 298 K): δ (ppm): 18.33. Raman (powder): ν/cm^{-1}: 1164 {s; expected ν(^{15}N^{15}N) stretch ≈ ν(^{14}N^{14}N) x [μ(^{14}N^{14}N)/μ(^{15}N^{15}N)]$^{0.5}$ = 1160 cm^{-1}}. All other spectroscopic data were consistent with **4**.

Synthesis of [{(Tren^{TMS})Ti^{IV}}$_2$(μ-η^1:η^1-N$_2$)][K(B15C5)$_2$]$_2$ (5)

At room temperature, and under a flow of N_2, B15C5 (0.564 g, 2.1 mmol) in toluene (40 mL) was slowly added *via* cannula to a rapidly stirring toluene solution of **4** (0.461 g, 0.5 mmol, 0.01 M). After 10 minutes, the resulting purple solution was concentrated *in vacuo* until saturated, then slowly cooled to –30 °C. Dark red crystals of **5** were formed overnight, which were collected by filtration, washed with pentane (3 x 20 mL), and then dried *in vacuo*. Yield: 531 mg, 53%. Anal. calcd. for $C_{86}H_{158}K_2N_{10}O_{20}Si_6Ti_2$: C 51.78; H 7.98; N 7.02%; Found: C 46.27; H 6.22; N 5.36%. (A satisfactory elemental analysis could not be obtained for this compound, possibly due to incomplete combustion.) ^{1}H NMR (C_6D_6, 298 K): δ (ppm): 6.82 (br s, 10H, crown-C_6H_4), 6.51 (br s, 10H, crown-C_6H_4), 3.56 (br s, overlap, tren-CH_2), 3.47 (br s, overlap, crown-CH_2), 2.66 (br s, 12H, tren-CH_2), 0.75 (s, 54H, SiMe_3). ^{13}C{^{1}H} NMR (C_6D_6, 298 K): δ (ppm): 121.90 (s, crown-C_6H$_4$), 114.13 (br s, crown-C_6H$_4$), 69.25 (br s, crown-CH$_2$), 62.40 (s, tren-CH$_2$), 48.96 (s, tren-CH$_2$), 5.15 (s, SiMe_3). ^{29}Si{^{1}H} NMR (C_6D_6, 298 K): δ (ppm): –5.35. Raman [powder; ν(N_2)]: ν/cm^{-1}: 1246 (br, s). ATR-FTIR (powder): ν/cm^{-1}: 2932 (s), 2871 (s), 2810 (s), 1596 (w), 1503 (s), 1455 (s), 1409 (w), 1362 (w), 1334 (w), 1302

(w), 1233 (s), 1123 (s), 1107 (s), 1095 (s), 1078 (s), 1057 (s), 1046 (s), 982 (s), 937 (s), 909 (s), 819 (s), 777 (s), 739 (s), 677 (s), 644 (s), 604 (w), 549 (s), 446 (s), 424 (s). UV/Vis: λ_{max}/nm (ε/$M^{-1}cm^{-1}$): C_6H_6, 314 (35,090), 398 (4,517); THF, 310 (25,855), 398 (3,071).

Synthesis of [{(Tren^{TMS})Ti}$_2$(μ-η^1:η^1-$^{15}N_2$)][K(B15C5)$_2$]$_2$ (5-$^{15}N_2$)

5-$^{15}N_2$ (admixed with **5**) was prepared from a mixture of **4** and **4-$^{15}N_2$** according to the procedure for **5**. ^{15}N NMR (C_6D_6, 298 K): δ (ppm): 18.61. Raman [powder; $\nu(N_2)$]: ν/cm^{-1}: 1203 {br, s; expected $\nu(^{15}N^{15}N)$ stretch $\approx \nu(^{14}N^{14}N$ x $[\mu(^{14}N^{14}N)/\mu(^{15}N^{15}N)]^{0.5}$ = 1204 cm^{-1}}. All other spectroscopic data were consistent with **5**.

***In situ interconversion* of 4 and Ti(Tren^{TMS})(I) (1B) *via* oxidation and reduction reactions under N_2**

1B was prepared *in situ* through oxidation of **4** with I_2 (1:2) in C_6D_6; subsequent reduction with KC_8 (4 eq.) reafforded **4**. Single crystal X-ray diffraction measurements for **1B** were performed on a yellow crystal obtained by slow cooling a pentane solution of **1B** to –30 °C. ^1H NMR (C_6D_6, 298 K): δ (ppm): 3.39 (t, 6H, CH_2), 2.10 (t, 6H, CH_2), 0.39 (s, 27H, CH_3). $^{29}Si\{^1H\}$ NMR (C_6D_6, 298 K): δ (ppm): 2.78 [$Si(CH_3)_3$].

Acidification Reactions

Acidification procedure under $^{14}N_2$ atmosphere

Inside a N_2 glovebox, the titanium complex (0.005 mmol), the acid, and the reductant, were weighed into bulb A of the distillation apparatus shown in Figure S1, which was then sealed and transferred to a dual-bank vacuum and N_2 gas manifold. The solvent was added to bulb B of the distillation apparatus *via* syringe and then frozen in a LN_2 bath (–196 °C). After placing the entire distillation apparatus under a static vacuum, the LN_2 bath was transferred to bulb A, such that the frozen solvent could be thawed and distilled onto the frozen solids in bulb A. The distillation apparatus was then opened to N_2 (ca 1 bar) and the LN_2 bath replaced with a dry ice/acetone bath (–78 °C). Once the solvent had thawed, bulb A was sealed under N_2 and left to stir at –78 °C, followed by 15 h at room

temperature. After this time, HCl (2 M in Et_2O, 2 mmol) was added to bulb B *via* syringe and then both bulbs were frozen in separate LN_2 baths and the entire distillation apparatus placed under a static vacuum and then sealed. After thawing bulb A, the volatiles were distilled onto the frozen HCl in bulb B. A further base distillation was performed by first resubmerging bulb A in a LN_2 bath before the addition of aqueous KOH (30%, 4 mL) *via* syringe under a flow of N_2. With both bulbs frozen, the entire distillation apparatus was once again reevacuated, sealed, and the contents of bulb A then allowed to thaw and distil under a static vacuum onto the frozen excess HCl in bulb B. The distillation was performed with vigorous stirring for approximately 30 minutes, after which time both bulbs were sealed and bulb B was warmed to room temperature and stirred for 10 minutes. All volatiles in bulb B were subsequently removed *in vacuo* and the remaining residue analysed for NH_3/NH_4Cl and N_2H_4/$N_2H_4{\cdot}2HCl$ *via* NMR spectroscopy and/or the indophenol and pdmab methods (*vide infra*). As previously reported,[6] N_2H_4 is only partially transferred under these conditions, and heating the distillation mixture should be avoided since N_2H_4 undergoes thermal decomposition to NH_3, N_2, and H_2. Thus, after the distillation, the solids remaining in bulb A were also analysed for N_2H_4/$N_2H_4{\cdot}2HCl$ *via* the pdmab method.

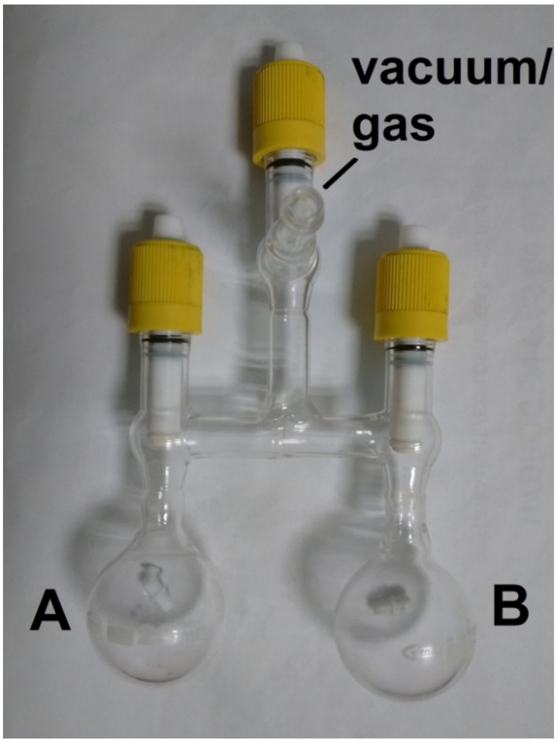

Figure S1. Glass distillation apparatus.

Acidification procedure under $^{15}N_2$ atmosphere

To exclude $^{14}N_2$ after the initial weighing, manipulations were performed under Ar utilising a dual-bank vacuum and Ar gas manifold; a Toepler line was used to deliver $^{15}N_2$ gas. Accordingly, inside a N_2 glovebox, the ^{15}N-isotopically labelled catalyst, **4-$^{15}N_2$**, the acid, and the reductant, were weighed into bulb A of the distillation apparatus, which was then sealed and transferred to a dual-bank vacuum and Ar gas manifold. The entire apparatus was degassed of N_2 (from the glovebox atmosphere), which was replaced with Ar over 3 cycles. Bulb B was charged with Ar-saturated solvent *via* syringe, then sealed and submerged in a LN_2/pentane bath (–131 °C). After placing the entire distillation apparatus under a static vacuum, the LN_2/pentane bath was transferred to bulb A, such that the frozen solvent could be thawed and distilled onto the frozen solids in bulb A. With the contents of bulb A frozen and the headspace evacuated, the distillation apparatus was connected to a Toepler line and bulb A placed under a $^{15}N_2$ atmosphere (1 bar, 780 eq.) and sealed. The distillation apparatus was then disconnected from the Toepler line and bulb A submerged in a dry ice/acetone bath. Once thawed, the mixture was stirred at –78 °C for 2 h then left to warm to room temperature and stir for a further 15 h. Subsequent distillation and analysis was performed as per the N_2 procedure.

NH_3 and N_2H_4 quantification procedures

The non-volatile residue remaining in bulb B contained any NH_3 and N_2H_4 (trapped as the conjugate acids NH_4Cl and $N_2H_4{\cdot}2HCl$) liberated from the reaction mixture *via* the distillation step. After dissolution in deionized H_2O (typically 5 mL), aliquots were taken for the indophenol and pdmab tests, and for NMR spectroscopic analysis. The remaining reaction mixture in bulb A contained any undistilled N_2H_4 as well as any reduced nitrogen fragments still bound to Ti; thus, after dissolution in aqueous HCl to make up a 1 M solution (10 mL after filtration), aliquots were analysed (after neutralisation) by the indophenol and pdmab tests. In all cases, only sub-stoichiometric quantities of N_2H_4/$N_2H_4{\cdot}2HCl$ and trace quantities of NH_3/NH_4Cl were detected. *Indophenol test (NH_3):* indophenol was generated *in situ* from NH_3 using an aqueous indicator solution of PhOH, NaOH, NaOCl, and $Na_2[Fe(CN)_5NO]$.[7] The concentration of NH_3 was determined *via* UV-vis spectroscopy by measuring the characteristic electronic absorption feature at 634 nm (Figure S2). *Pdmab test*

(N_2H_4): a yellow azine dye was generated *in situ* from N_2H_4 using an acidic indicator solution of *para*-dimethylaminobenzaldehyde (pdmab) in EtOH.[8] The concentration of N_2H_4 was determined *via* UV-vis spectroscopy by measuring the characteristic electronic absorption feature at 458 nm (Figure S3).

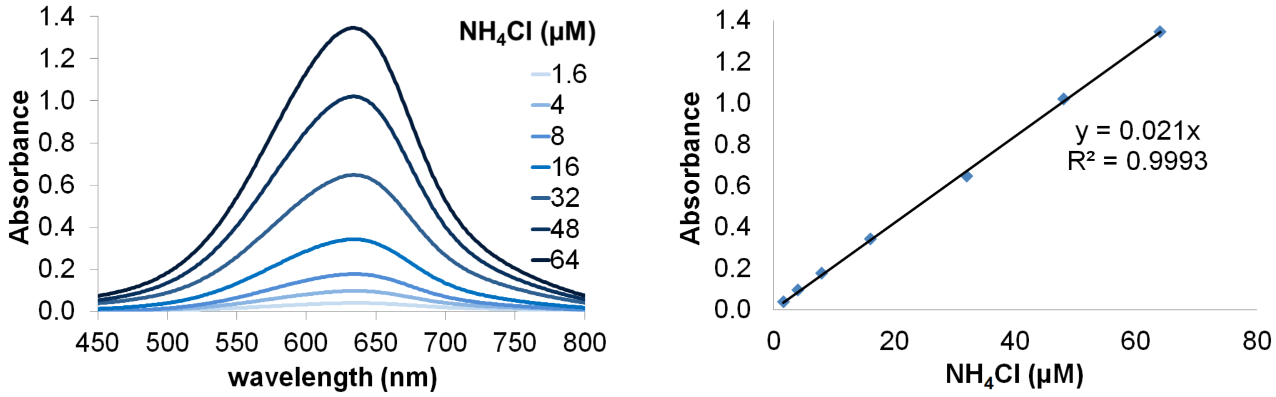

Figure S2. UV-vis calibration curve for the indophenol method (λ = 634 nm).

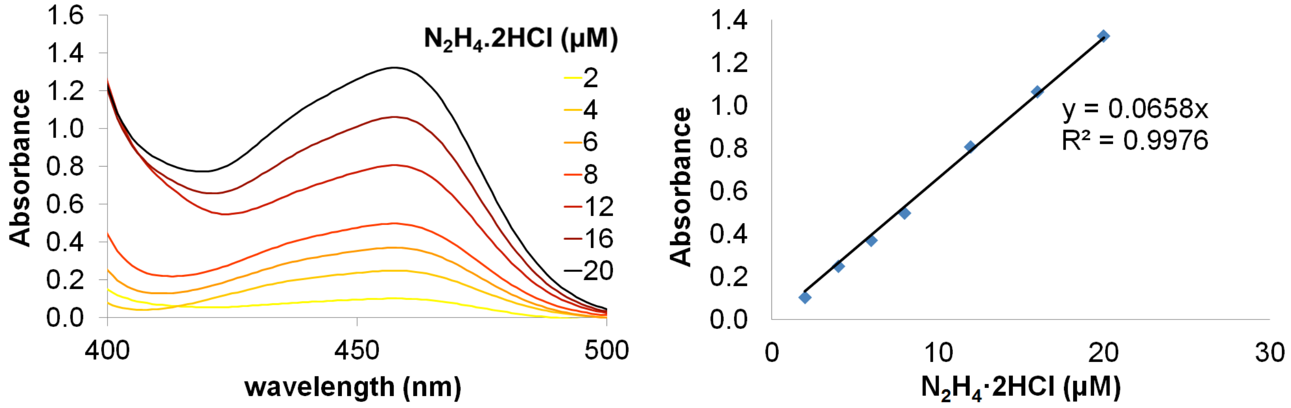

Figure S3. UV-vis calibration curve for the pdmab hydrazine test (λ = 458 nm).

^{1}H NMR spectroscopy (NH_3): An aliquot taken from the aqueous analyte was dried *in vacuo* and then dissolved in DMSO-d_6 (0.7 mL) for ^{1}H NMR spectroscopic analysis (delay time = 25 s; Figure S4-6). The $^{14}NH_4^+$ 1:1:1 triplet (7.28 ppm, $^1J_{NH}$ = 51 Hz) or $^{15}NH_4^+$ 1:1 doublet (7.28 ppm, $^1J_{NH}$ = 71 Hz) was integrated relative to the methyl protons of 2,5-dimethylfuran (2.14 ppm, s),[6] contained within a DMSO-d_6 sealed capillary insert and calibrated using a standard 0.16 M solution of NH_4Cl in DMSO-d_6.

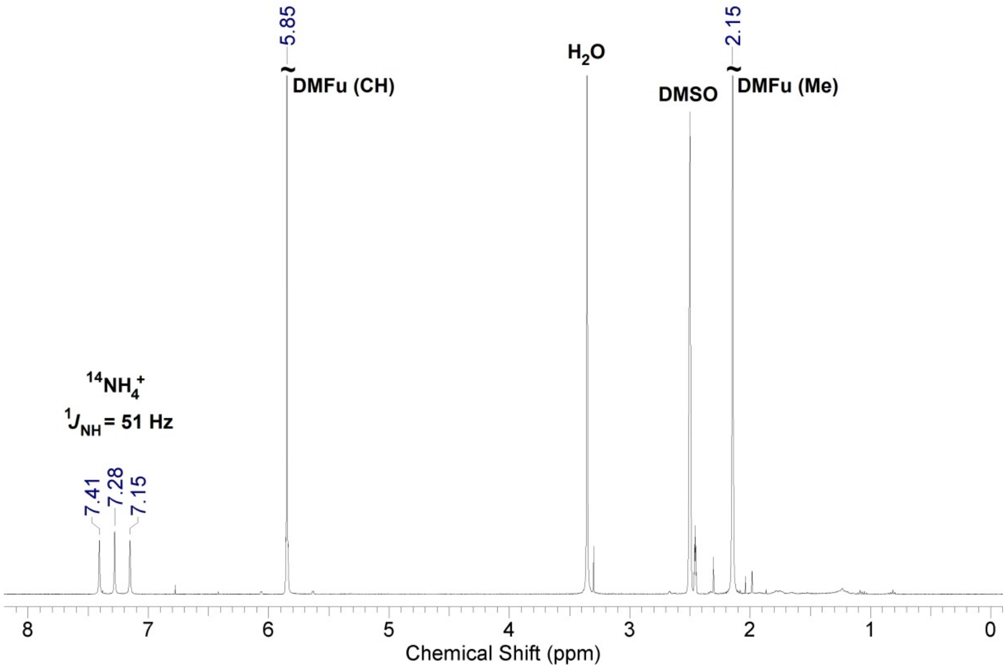

Figure S4. ^{1}H NMR spectrum of an aliquot taken from the HCl-trapped volatile fraction in a catalytic acidification reaction (Table 1, Entry 6), dissolved in DMSO-d$_6$. The $^{14}NH_4^+$ 1:1:1 triplet (7.28 ppm, $^{1}J_{NH}$ = 51 Hz) was integrated relative to the methyl protons of 2,5-dimethylfuran (2.15 ppm, s).

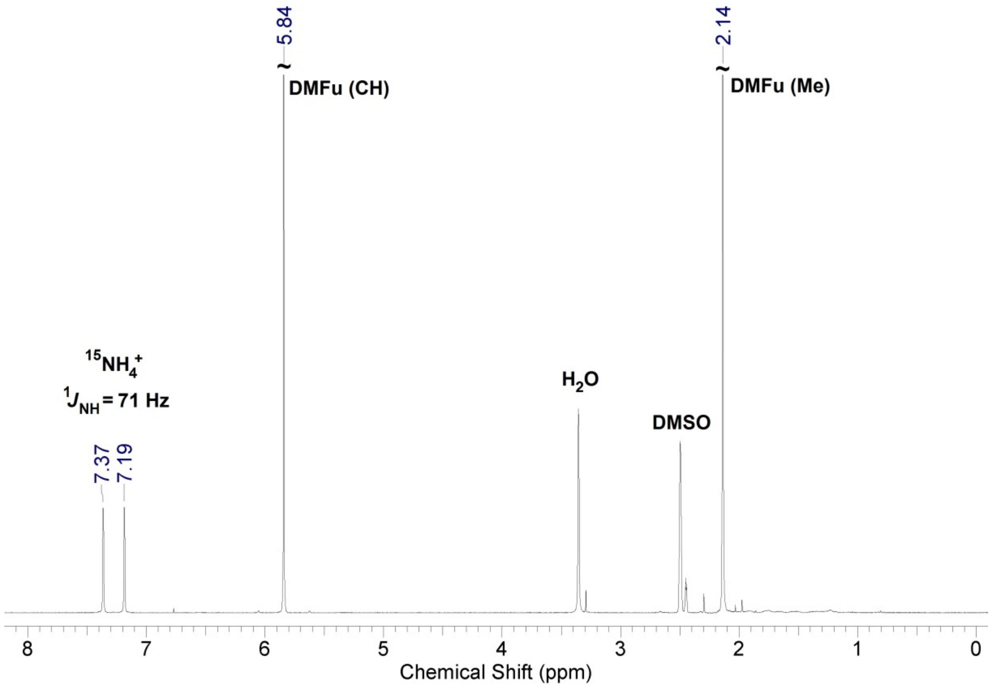

Figure S5. ^{1}H NMR spectrum of an aliquot taken from the HCl-trapped volatile fraction in a catalytic acidification reaction (Table 1, Entry 7), dissolved in DMSO-d$_6$. The $^{15}NH_4^+$ 1:1 doublet (7.28 ppm, $^{1}J_{NH}$ = 71 Hz) was integrated relative to the methyl protons of 2,5-dimethylfuran (2.15 ppm, s).

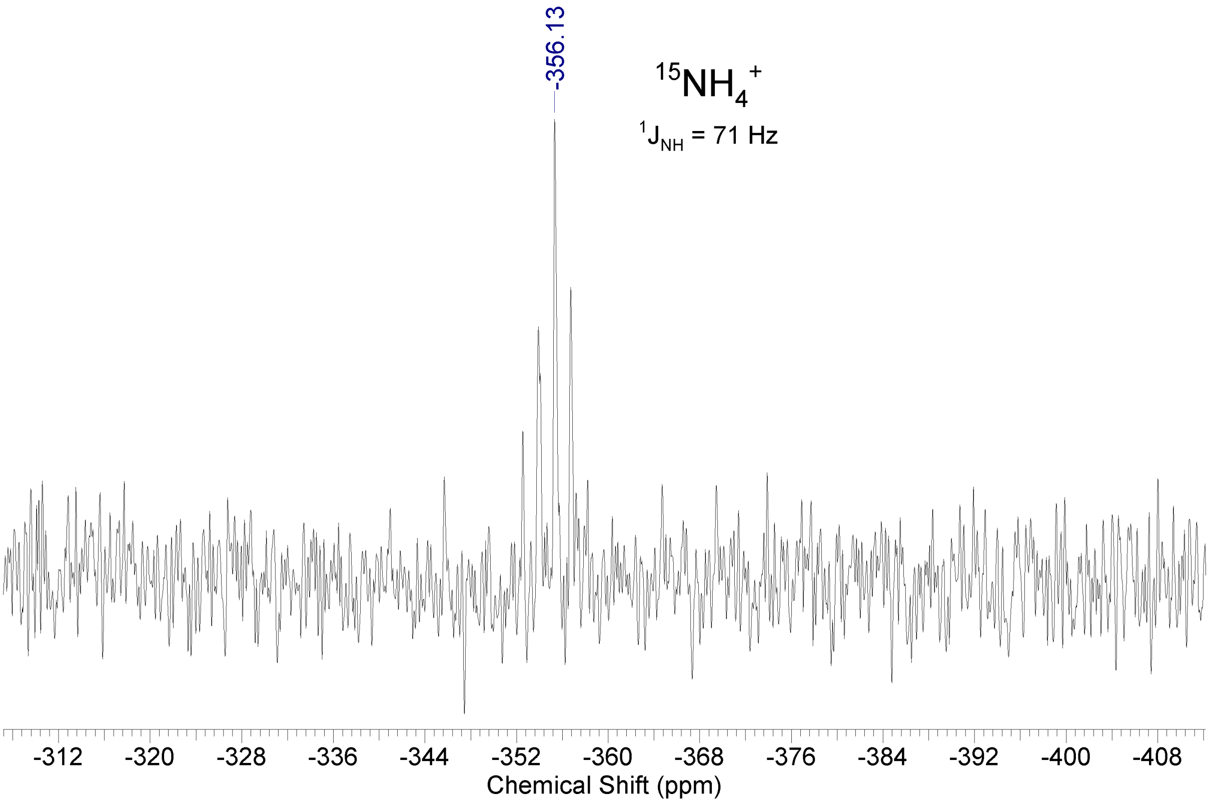

Figure S6. ^{15}N NMR spectrum of an aliquot taken from the HCl-trapped volatile fraction in a catalytic acidification reaction (Table 1, Entry 7), dissolved in DMSO-d_6. $^{15}NH_4^+$ appears as a 1:4:6:4:1 quintet (356.1 ppm, $^1J_{NH}$ = 71 Hz).

NMR spectra

[(Tren^{TMS})Ti^{IV}](I) (1B)

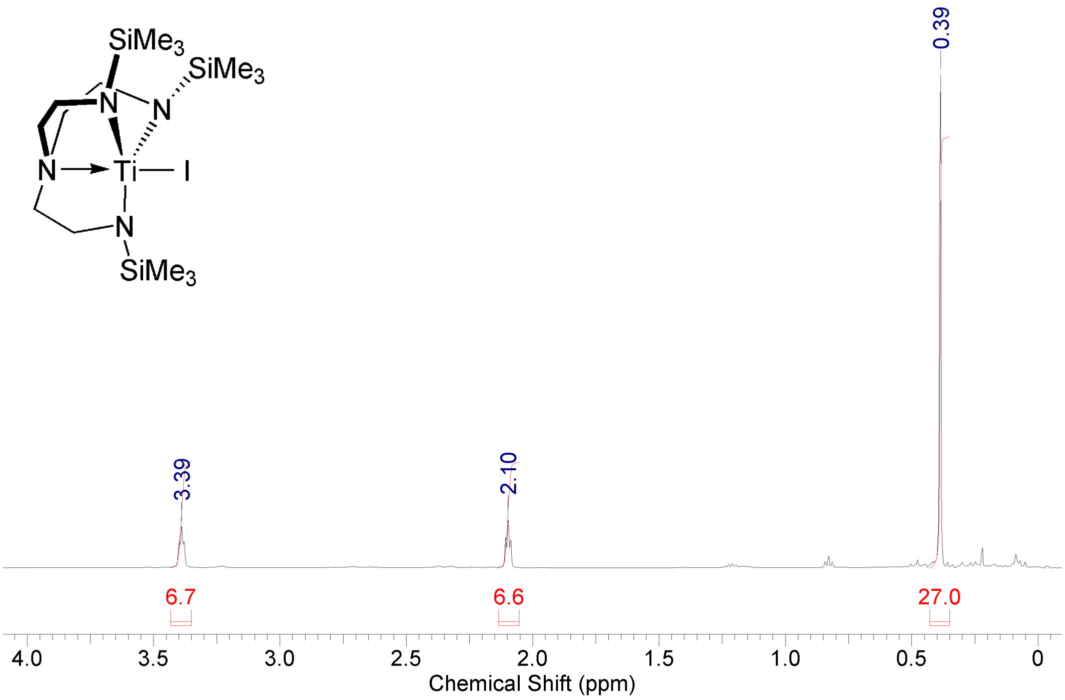

Figure S7. ^{1}H NMR spectrum of **1B** in C_6D_6.

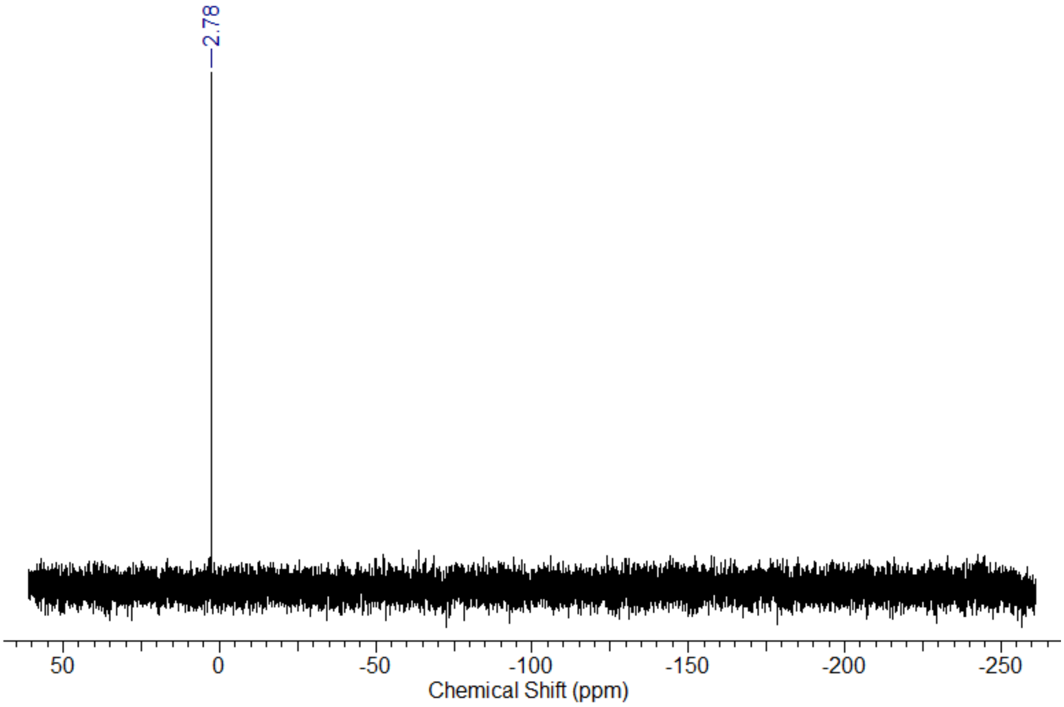

Figure S8. ^{29}Si{^{1}H} NMR spectrum of **1B** in C_6D_6.

[{(Tren^{TMS})Ti^{III}}$_2$(μ-η^1:η^1-N$_2$)] (3)

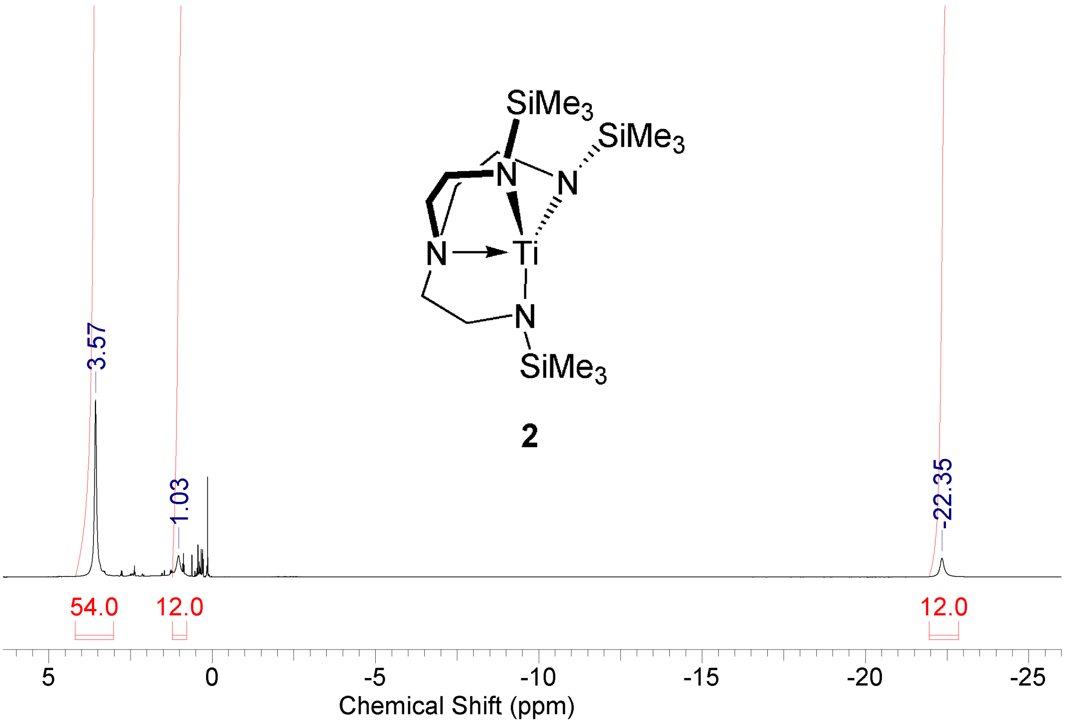

Figure S9. ^{1}H NMR spectrum of **3** (dissociated as **2**) in C_6D_6.

[{(Tren^{TMS})Ti^{IV}}$_2$(μ-η^1:η^1:η^2:η^2-N$_2$K$_2$)] (4)

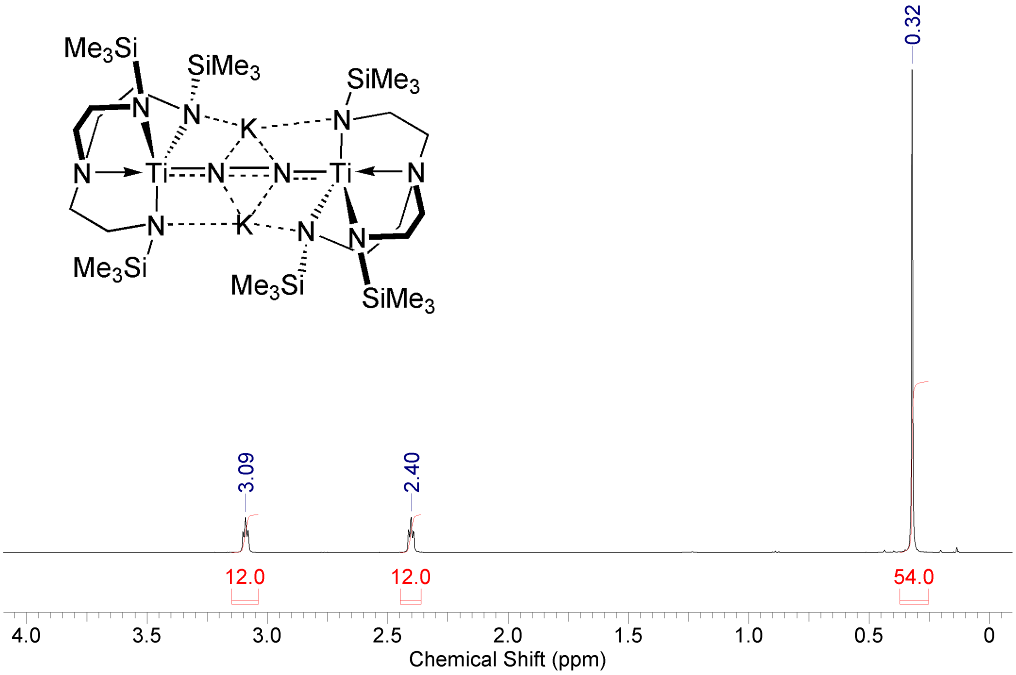

Figure S10. ^{1}H NMR spectrum of **4** in C_6D_6.

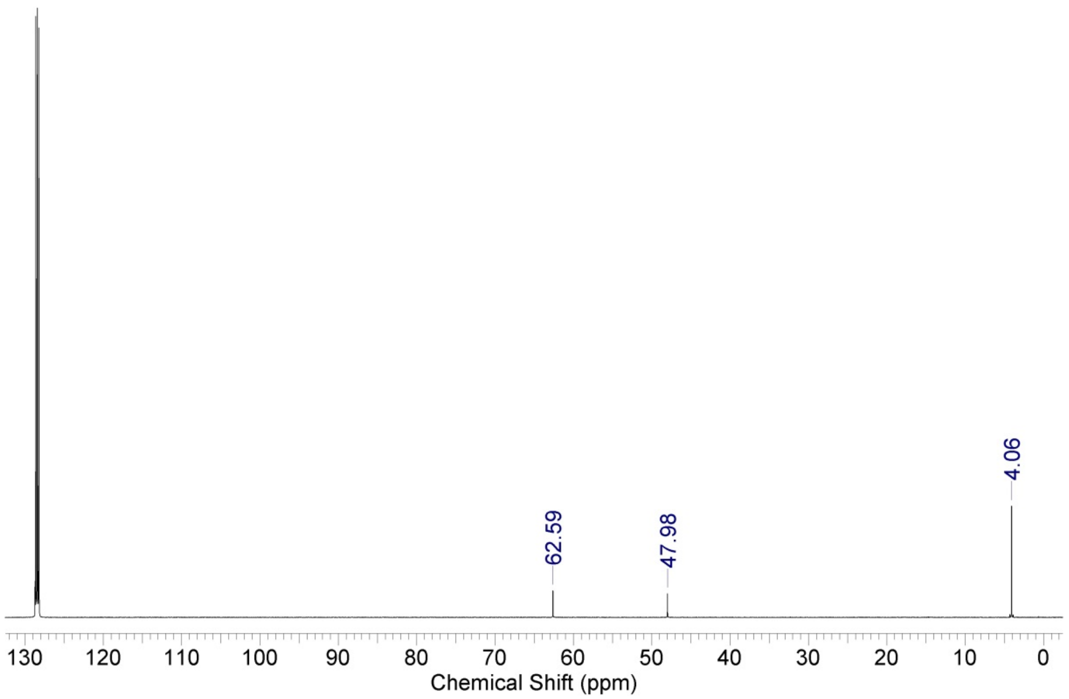

Figure S11. $^{13}C\{^{1}H\}$ NMR spectrum of **4** in C_6D_6.

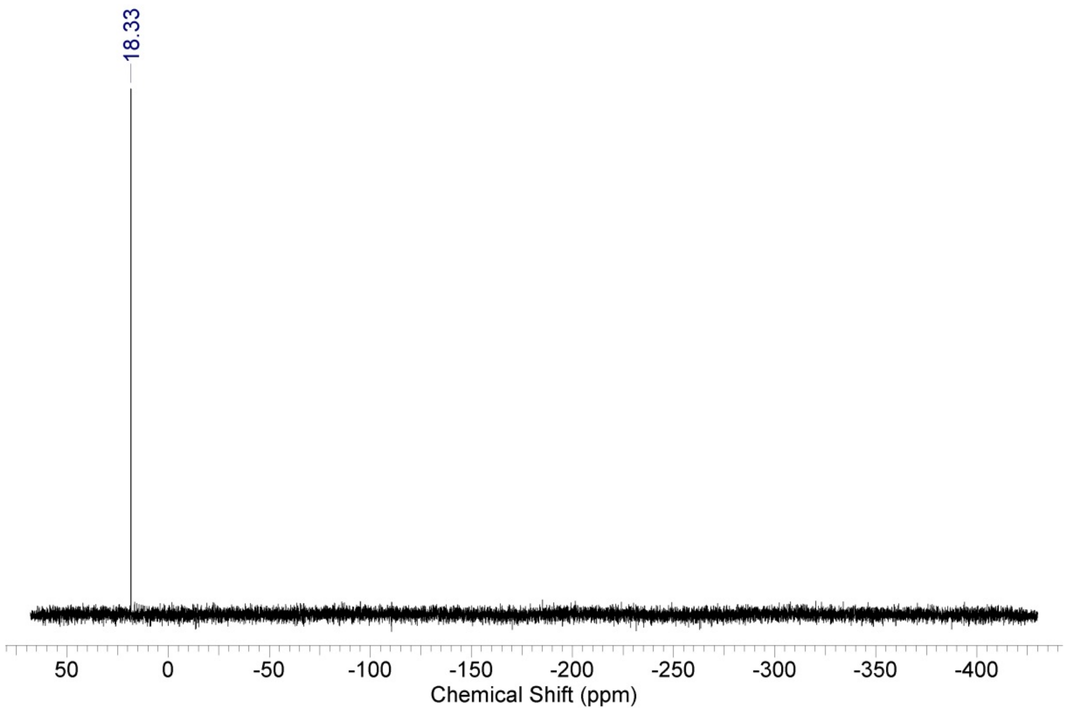

Figure S12. ^{15}N NMR spectrum of **4-^{15}N$_2$** in C_6D_6.

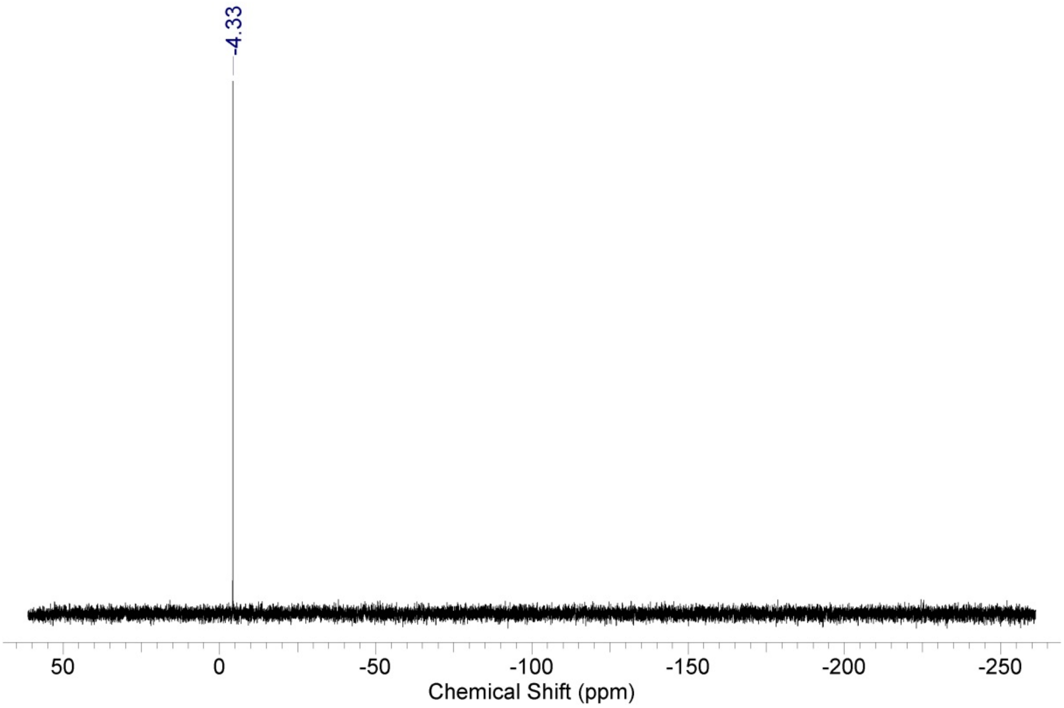

Figure S13. $^{29}Si\{^{1}H\}$ NMR spectrum of **4** in C_6D_6.

Synthesis of [{(Tren^{TMS})Ti^{IV}}$_2$(μ-η^1:η^1-N$_2$)][K(B15C5)$_2$]$_2$ (5)

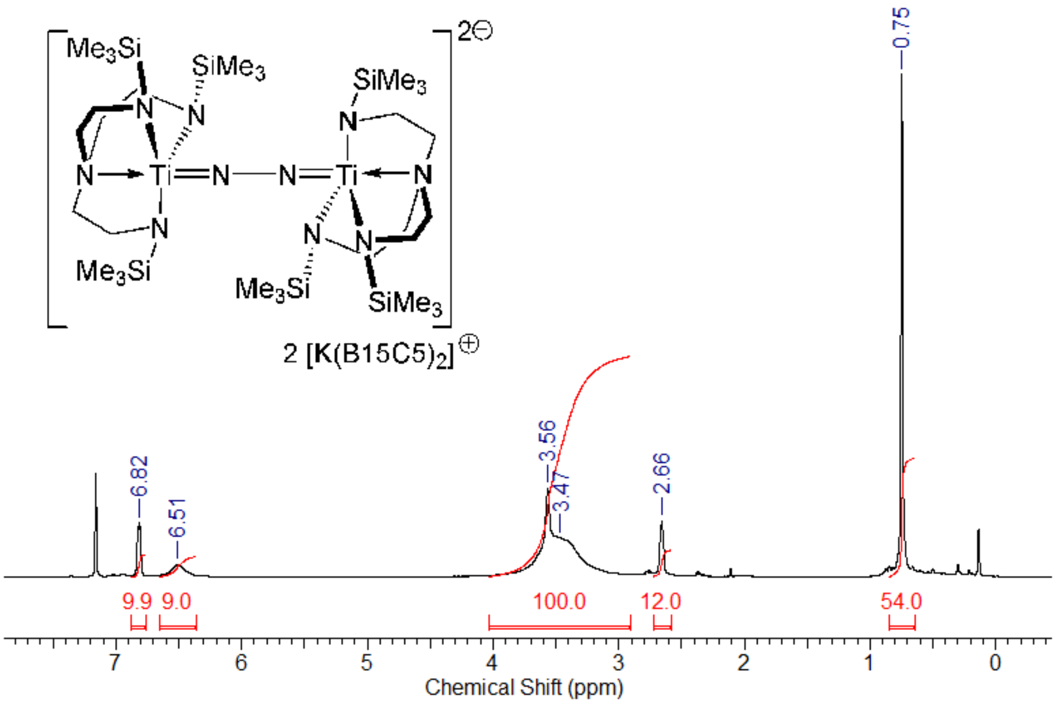

Figure S14. ^{1}H NMR spectrum of **5** in C_6D_6.

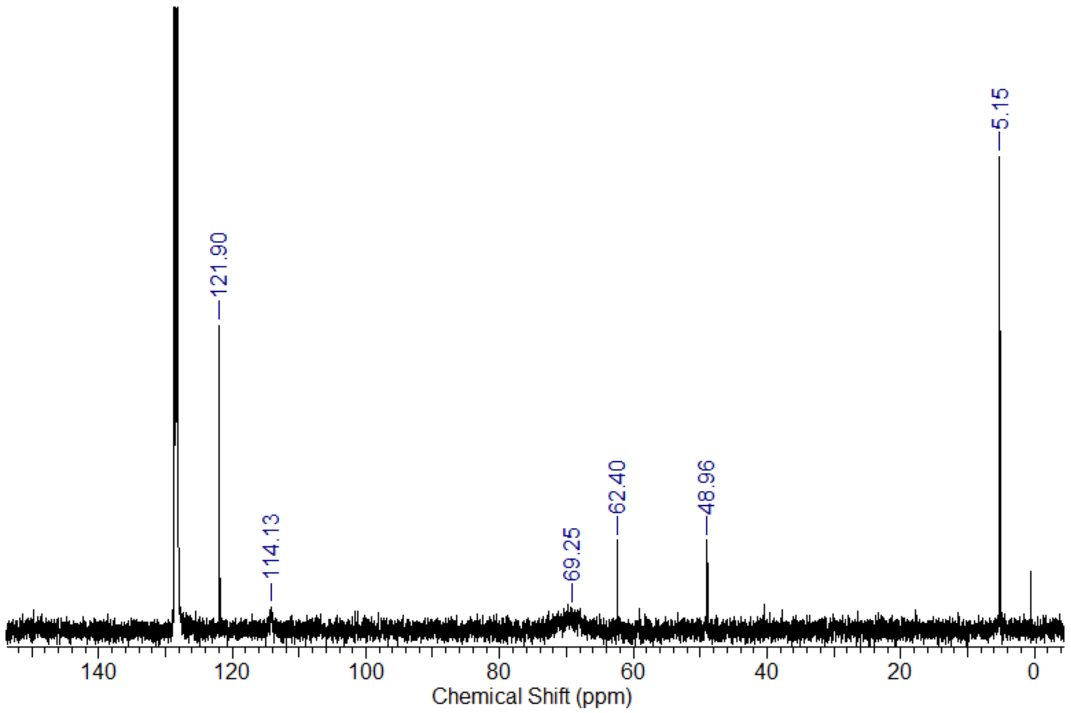

Figure S15. $^{13}C\{^{1}H\}$ NMR spectrum of **5** in C_6D_6.

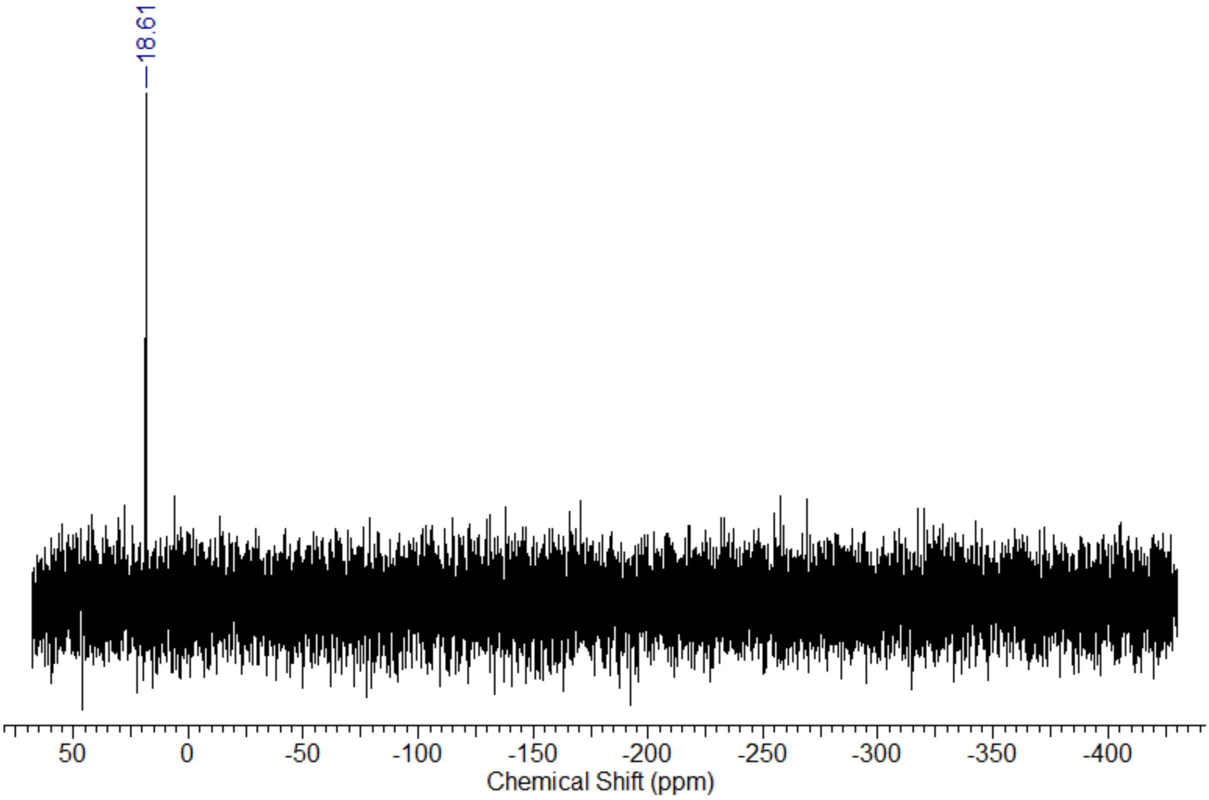

Figure S16. ^{15}N NMR spectrum of **5-$^{15}N_2$** in C_6D_6.

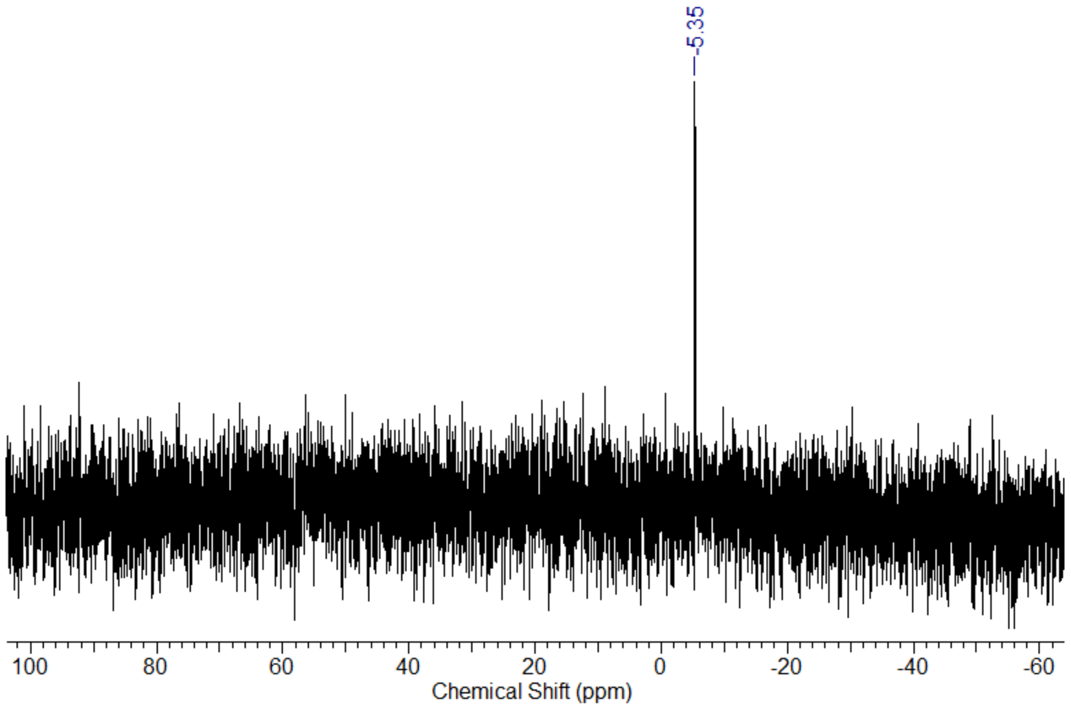

Figure S17. $^{29}Si\{^1H\}$ NMR spectrum of **5** in C_6D_6.

EPR spectra

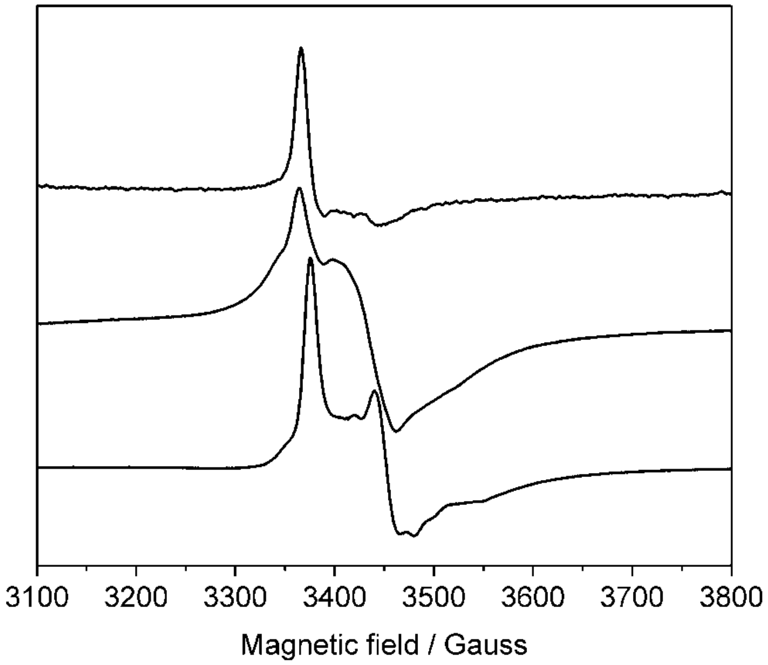

Figure S18. X-band EPR spectra of **3** as a powder at room temperature (top), at 20 K (middle), and of **2** as a frozen toluene-pentane solution at 20 K (bottom).

FTIR and Raman spectra

[{(Tren^{TMS})Ti^{III}}$_2$(μ-η^1:η^1-N$_2$)] (3)

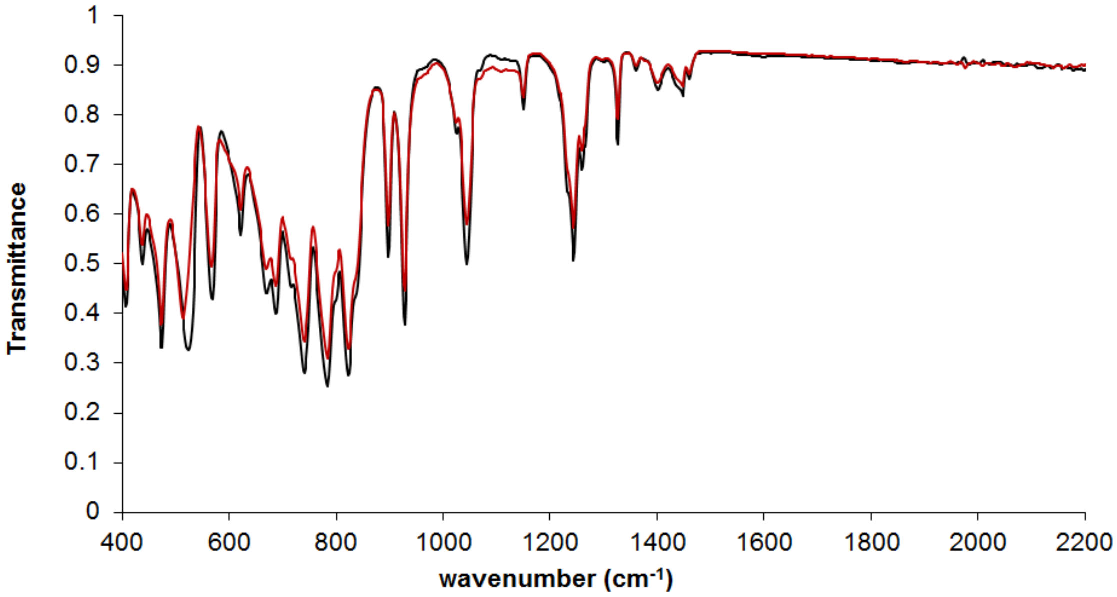

Figure S19. FTIR spectra of **3** (black trace) and **3-^{15}N$_2$** (admixed with **3**; red trace); recorded as powders.

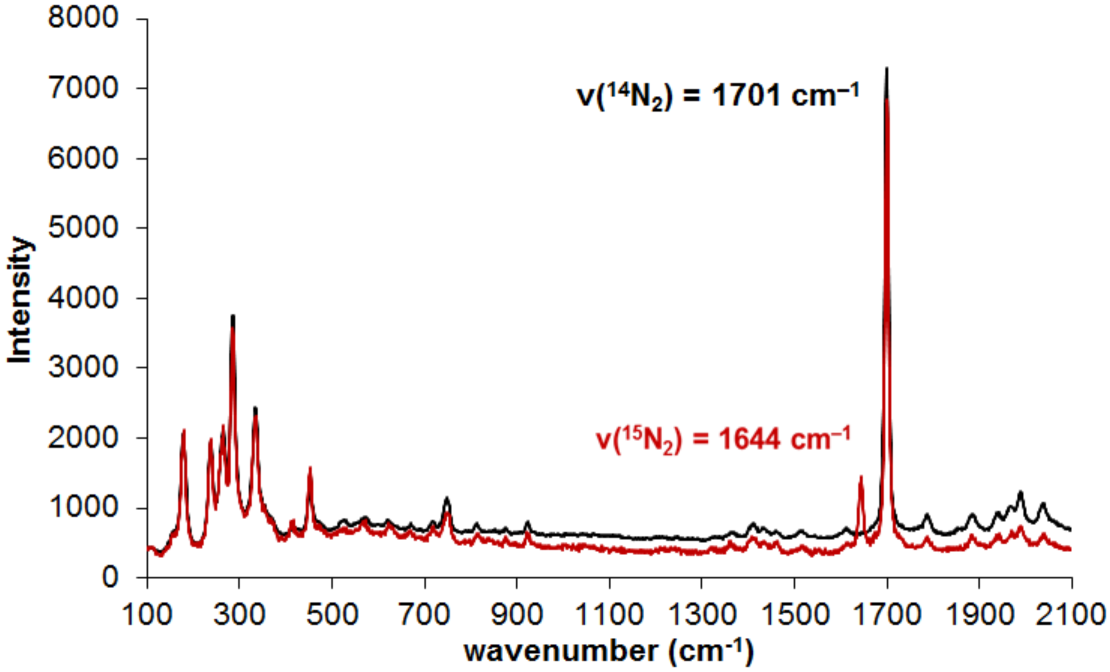

Figure S20. Raman spectra of **3** (black trace) and **3-^{15}N$_2$** (admixed with **3**; red trace); recorded as powders.

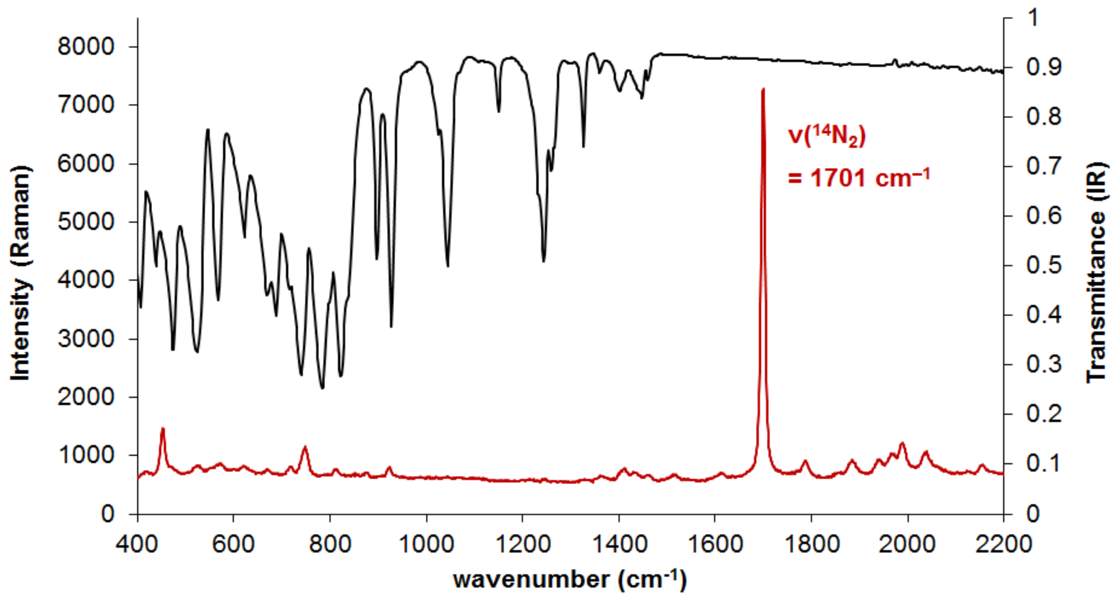

Figure S21. Overlaid FTIR (transmittance, black trace) and Raman (intensity, red trace) spectra of **3**; recorded as powders.

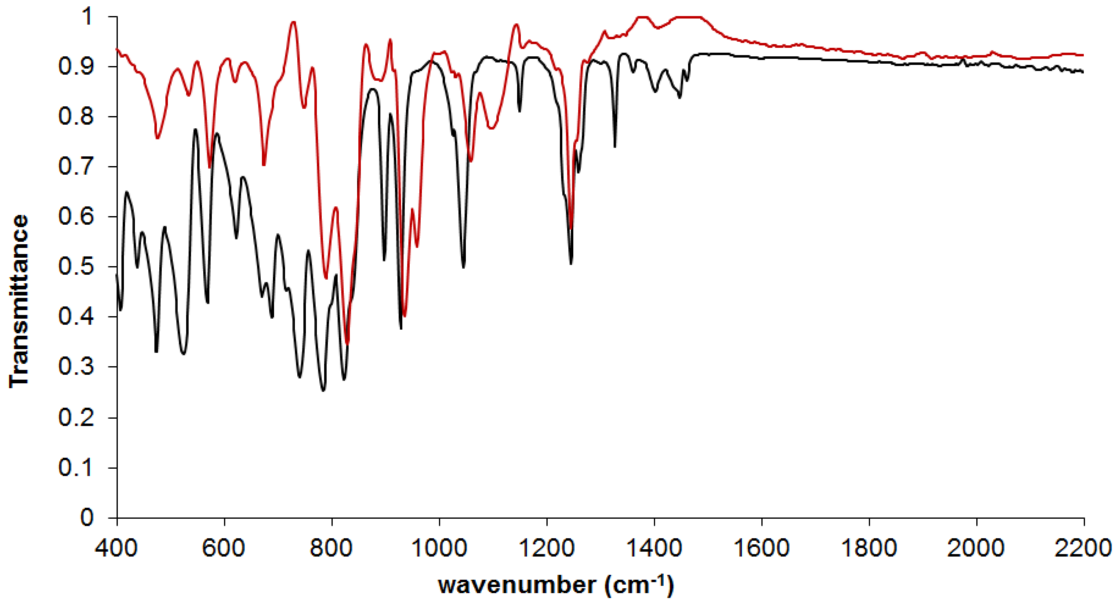

Figure S22. FTIR spectra of **3**; recorded as a powder (black trace) and as a 10 mM pentane solution (red trace).

$[\{(Tren^{TMS})Ti^{IV}\}_2(\mu\text{-}\eta^1:\eta^1:\eta^2:\eta^2\text{-}N_2K_2)]$ (4)

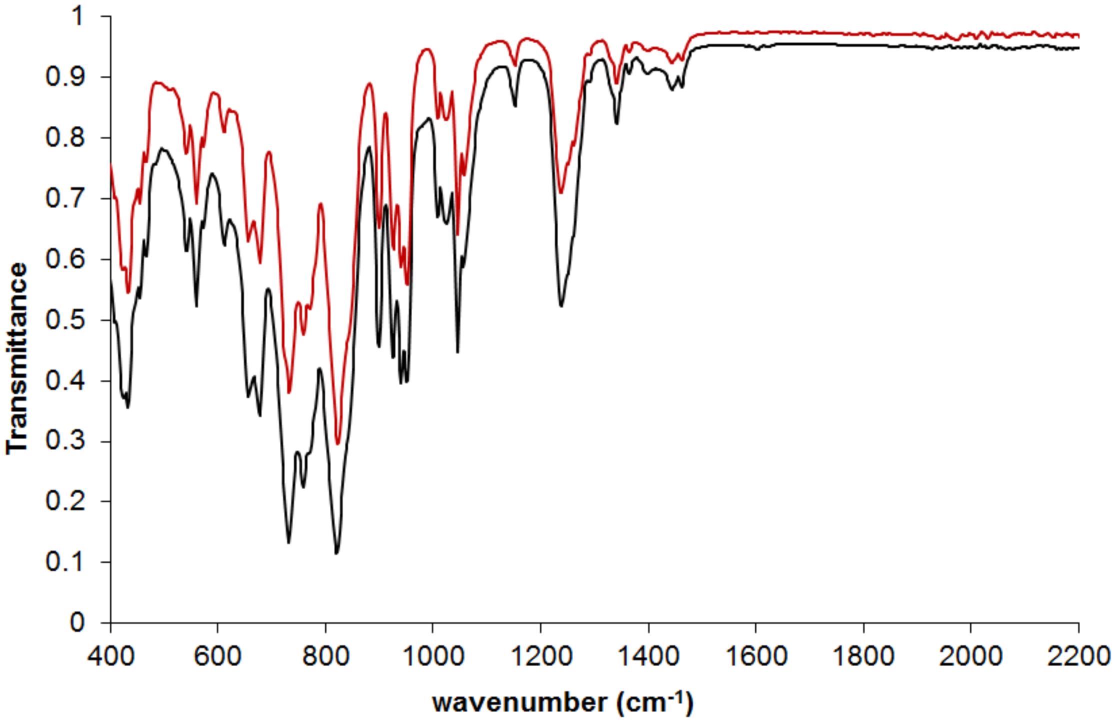

Figure S23. FTIR spectra of **4** (black trace) and **4-^{15}N$_2$** (red trace); recorded as powders.

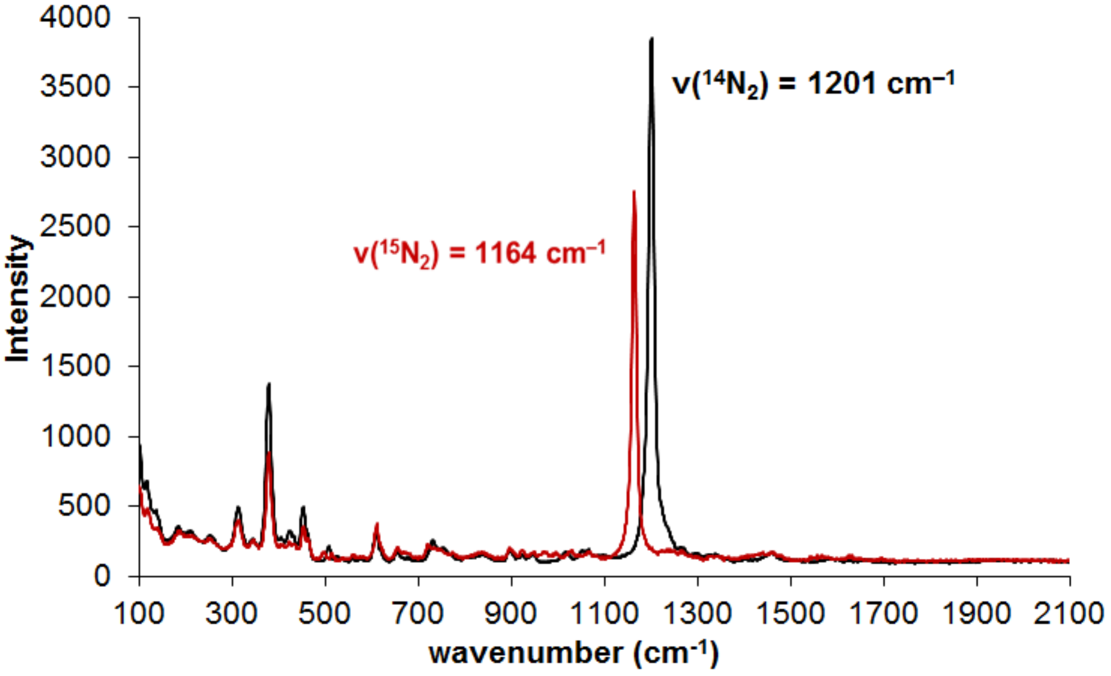

Figure S24. Raman spectra of **4** (black trace) and **4-^{15}N$_2$** (red trace); recorded as powders.

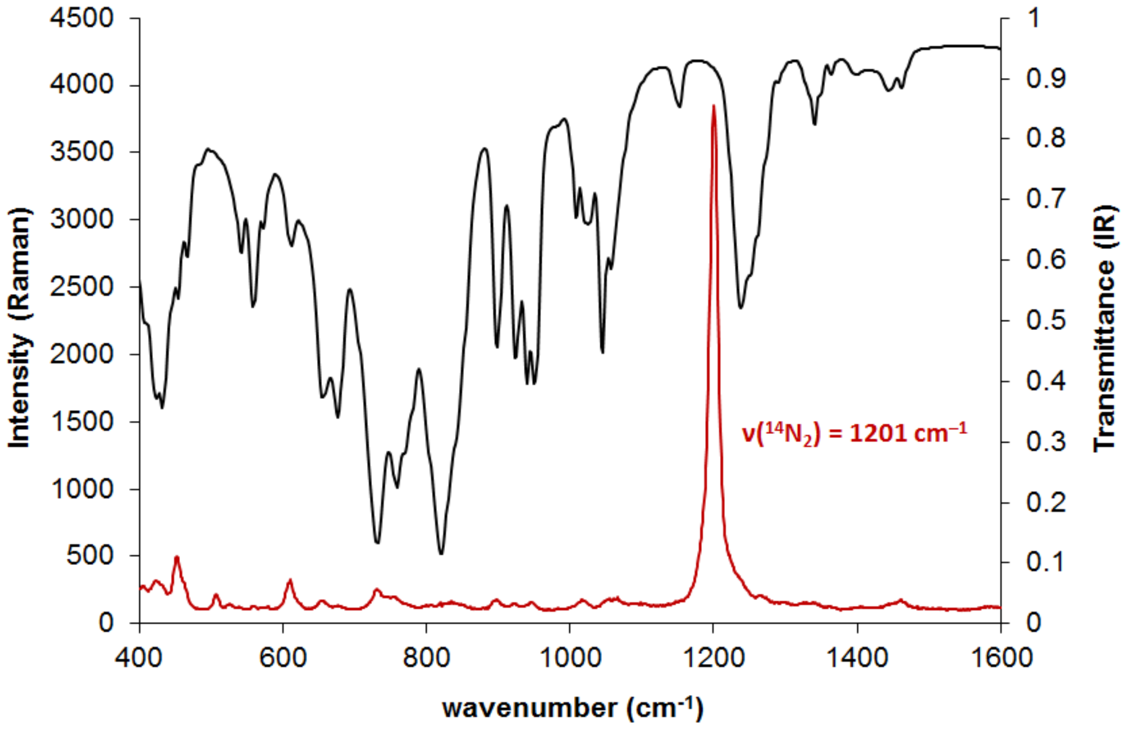

Figure S25. Overlaid FTIR (transmittance, black trace) and Raman (intensity, red trace) spectra of **4**; recorded as powders.

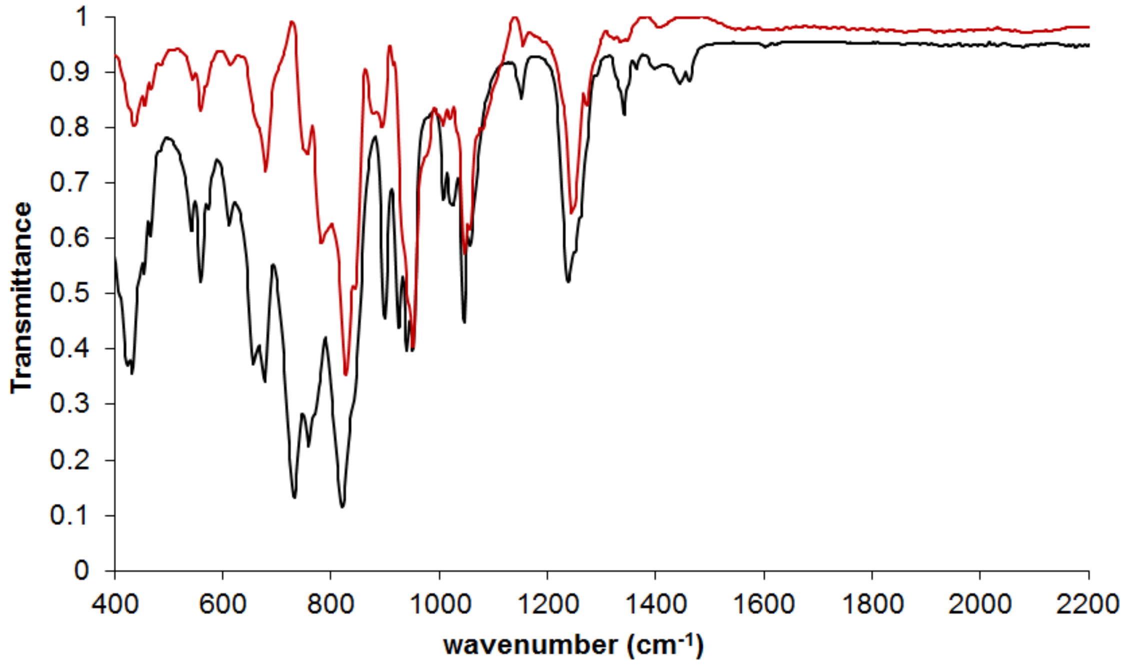

Figure S26. FTIR spectra of **4**; recorded as a powder (black trace) and as a 10 mM pentane solution (red trace).

[{(Tren^{TMS})Ti^{IV}}$_2$(μ-η^1:η^1-N$_2$)][K(B15C5)$_2$]$_2$ (5)

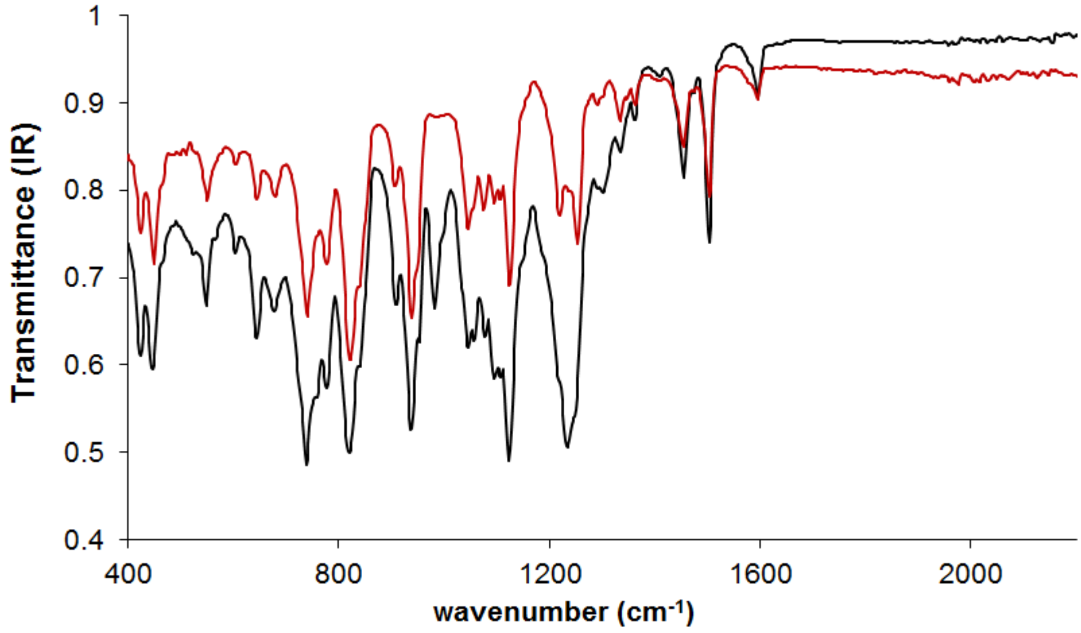

Figure S27. FTIR spectra of **5** (black trace) and **5**-^{15}**N$_2$** (admixed with **5**; red trace); recorded as powders.

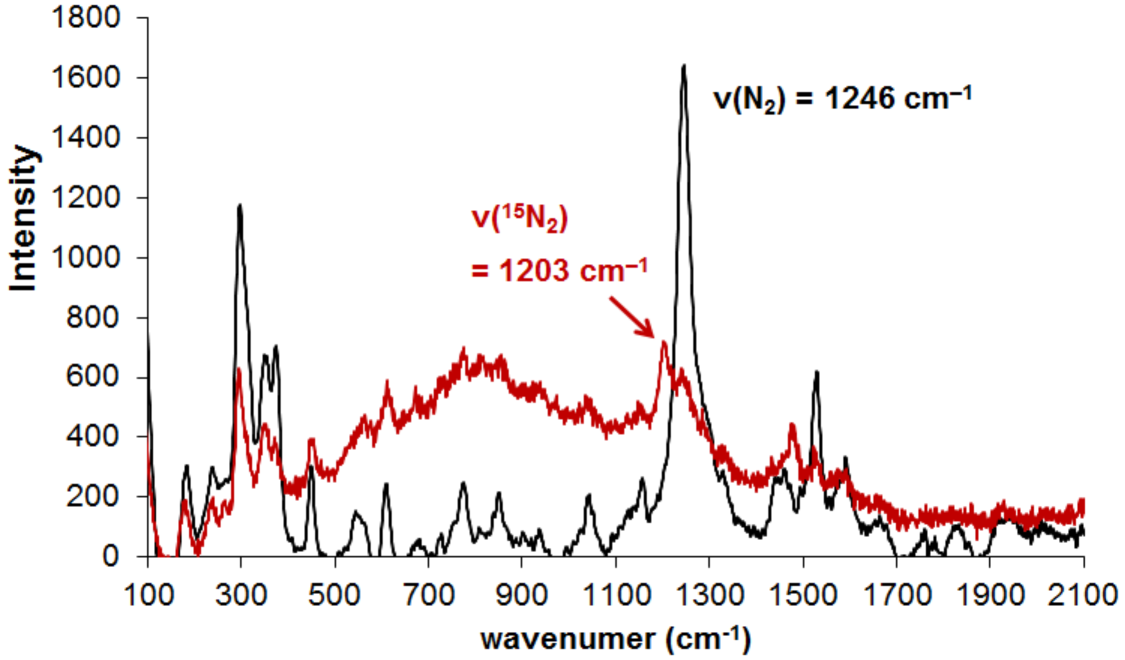

Figure S28. Raman spectra of **5** (black trace) and **5**-^{15}**N$_2$** (admixed with **2'**; red trace); recorded as powders.

UV-Vis spectra

[{(Tren^{TMS})Ti^{III}}$_2$(μ-η^1:η^1-N$_2$)] (3)

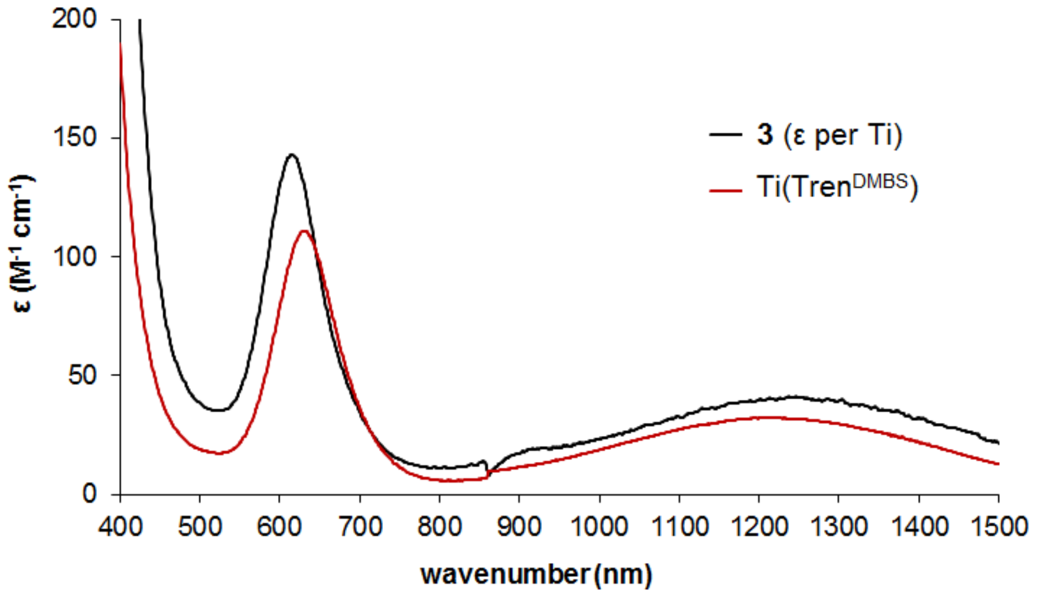

Figure S29. UV-vis spectra of **3** (black trace) and Ti(Tren^{DMBS}) (red trace) recorded in toluene; ε plotted per Ti centre.

[{(Tren^{TMS})Ti^{IV}}$_2$(μ-η^1:η^1:η^2:η^2-N$_2$K$_2$)] (4)

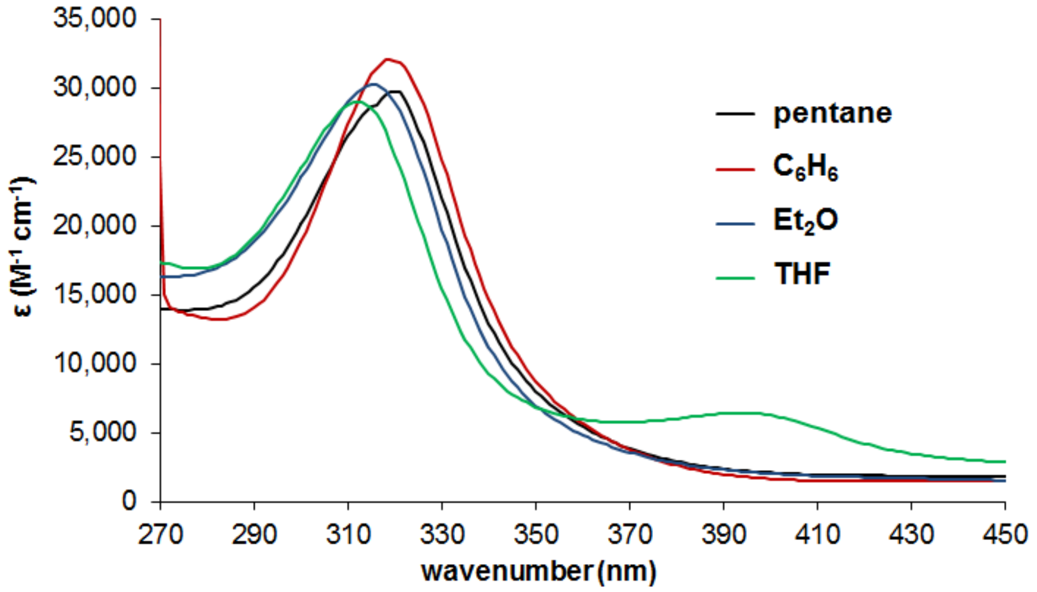

Figure S30. UV-vis spectra of **4** recorded in pentane (black trace), C_6H_6 (red trace), Et_2O (blue trace), and THF (green trace).

[{(Tren^{TMS})Ti^{IV}}$_2$(μ-η^1:η^1-N$_2$)][K(B15C5)$_2$]$_2$ (5)

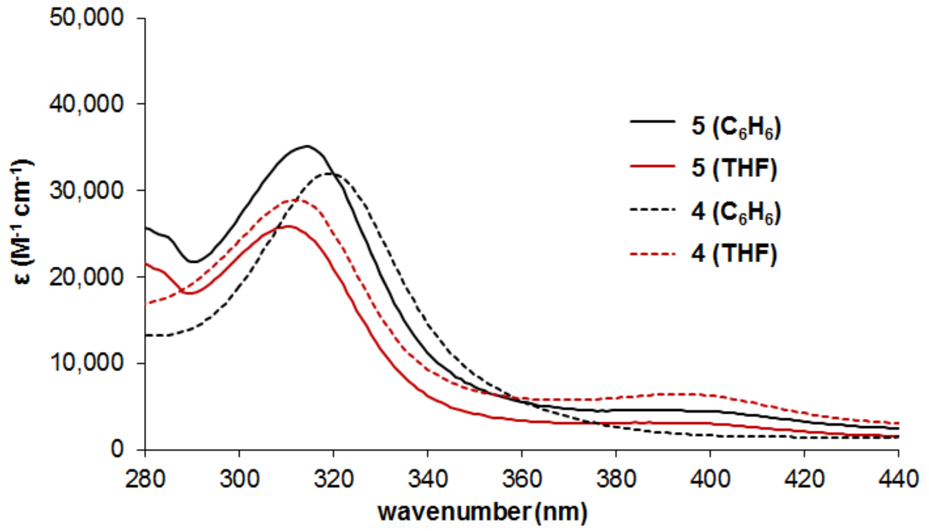

Figure S31. UV-vis spectra of **5** recorded in C_6H_6 (black solid trace) and THF (red solid trace); overlaid with **4** recorded in C_6H_6 (black dotted trace) and THF (red dotted trace)

SQUID data

[{(Tren^{TMS})Ti^{III}}$_2$(μ-η^1:η^1-N$_2$)] (3)

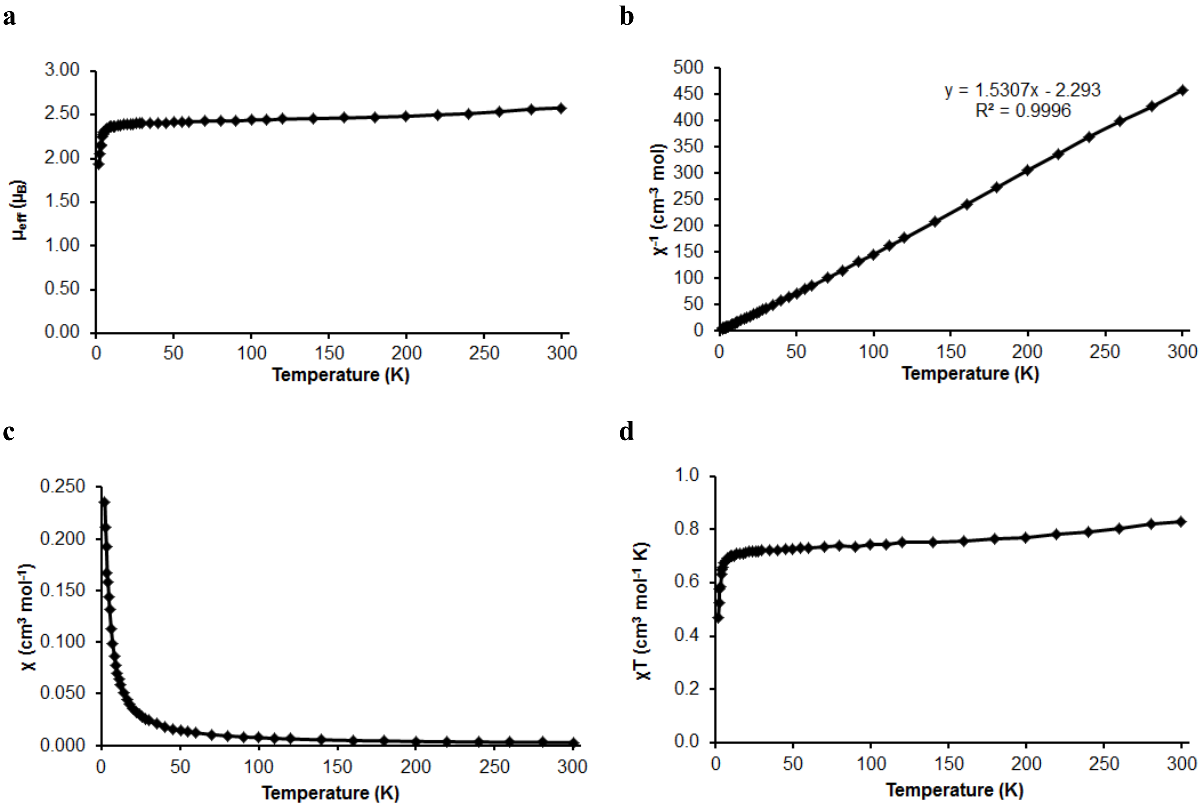

Figure S32. Magnetic susceptibility data for **3**: (a) μ_{eff} vs T; (b) χ^{-1} vs T; (c) χ vs T; (d) χT vs T.

X-ray diffraction data

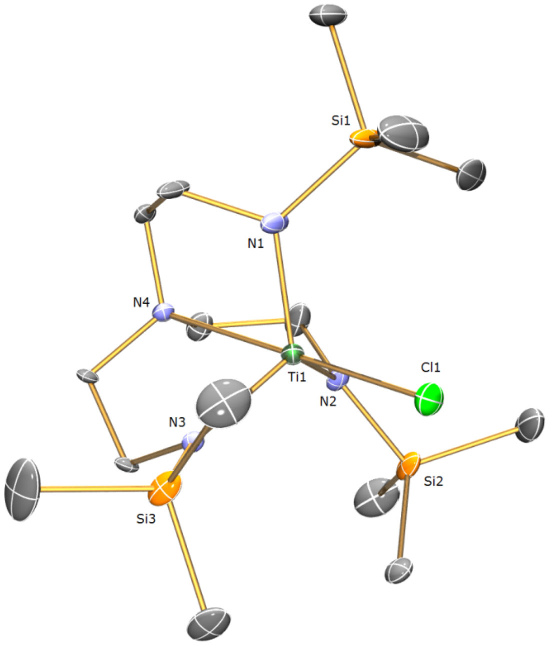

Figure S33. Molecular structure of **1A** (120K) with displacement ellipsoids at 50% probability. Hydrogen atoms and disordered components are omitted for clarity.

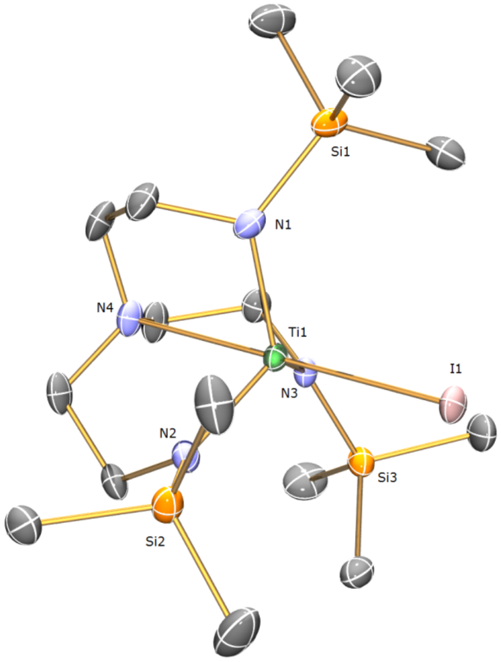

Figure S34. Molecular structure of **1B** (120K) with displacement ellipsoids at 50% probability. Hydrogen atoms and disordered components are omitted for clarity.

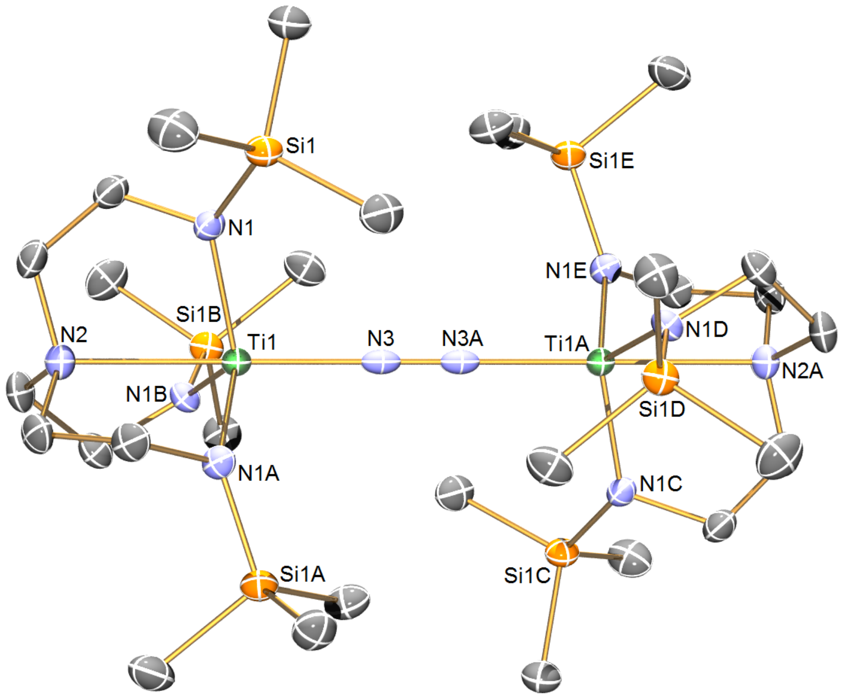

Figure S35. Molecular structure of **3** (150 K) with displacement ellipsoids at 50% probability. Hydrogen atoms are omitted for clarity.

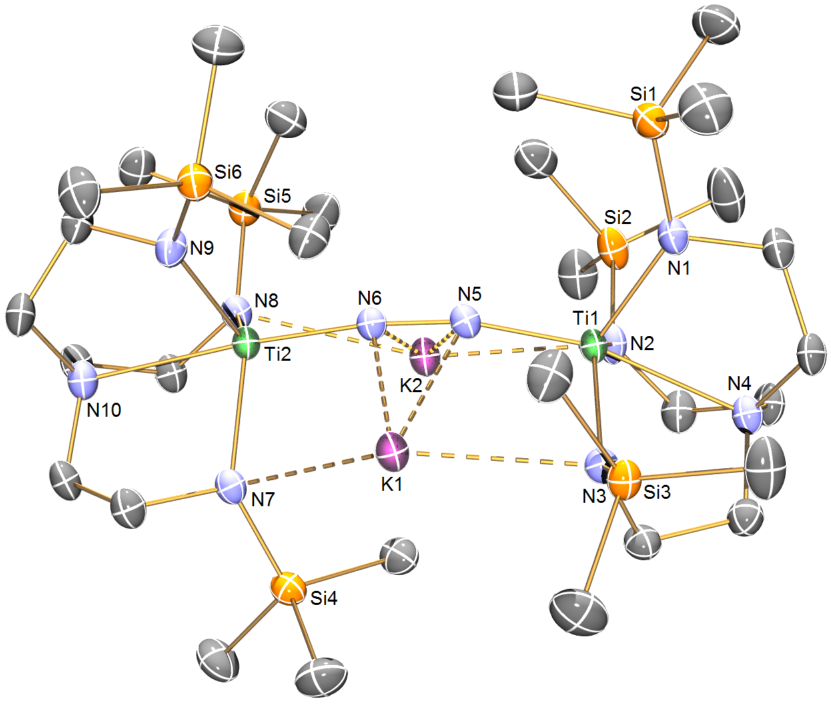

Figure S36. Molecular structure of **4** (120 K) with displacement ellipsoids at 50% probability. Hydrogen atoms are omitted for clarity.

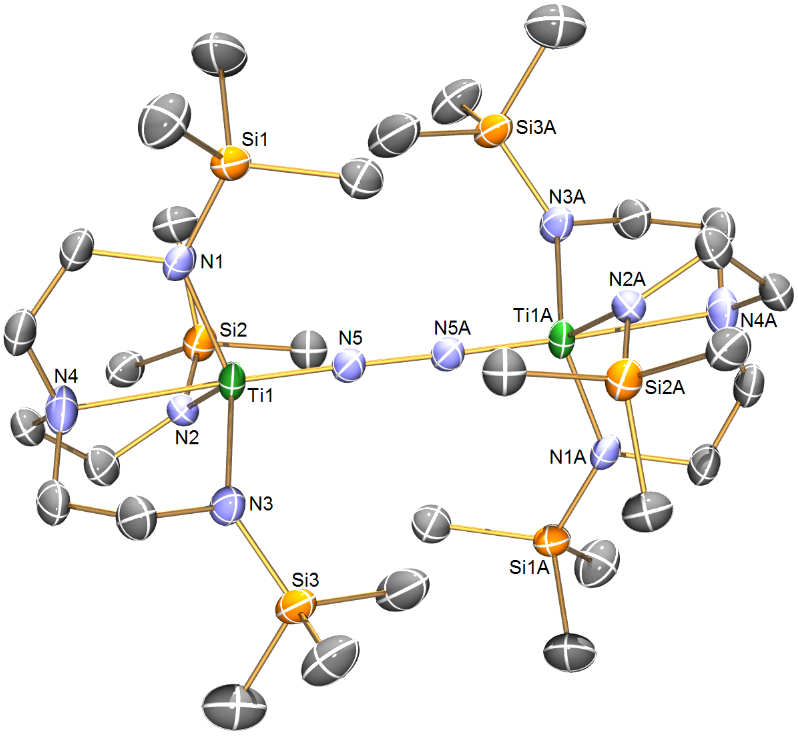

Figure S37. Molecular structure of **5** (150 K) with displacement ellipsoids at 50% probability. The two $[K(B15C5)_2]^+$ cations and hydrogen atoms are omitted for clarity.

Computational details

General

Unrestricted and restricted geometry optimisations were performed for the full models of **3-5** using initial coordinates derived from the X-ray crystal structures. No constraints were imposed on the calculations. The calculations were performed using the Amsterdam Density Functional (ADF) suite version 2012.01.[9,10] The DFT geometry optimisations employed Slater type orbital (STO) triple-ζ-plus polarisation all-electron basis sets (from the ZORA/TZP database of the ADF suite). Scalar relativistic approaches were used within the ZORA Hamiltonian for the inclusion of relativistic effects and the local density approximation (LDA) with the correlation potential due to Vosko et al[11] was used in all of the calculations. Gradient corrections were performed using the functionals of Becke[12] and Perdew.[13] MOLEKEL[14] was used to prepare the three-dimensional plot of the electron density. Analytical frequencies were computed within the ADF program.

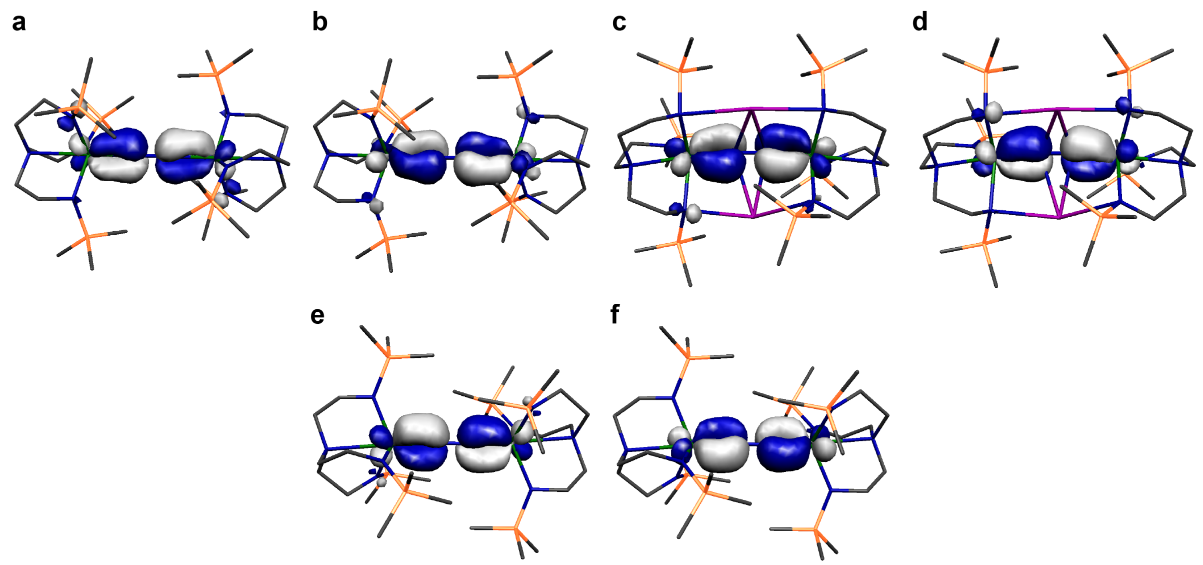

Figure S38. Kohn Sham Molecular Orbitals showing the principal Ti→N_2-π* pairs of back-bonding interactions of: a) HOMO of **3** (229a, −3.564 eV); b) HOMO−1 of **3** (228a, −3.567 eV); c) HOMO of **4** (247a, −3.123 eV); d) HOMO−1 of **4** (246a, −3.209 eV); e) HOMO of **5** (229a, 2.443 eV); f) HOMO−1 of **5** (228a, 2.387 eV). Hydrogen atoms are omitted for clarity. Colour key: titanium(green); carbon (gray); nitrogen (blue); silicon (orange); potassium (purple).

Table S1. Final coordinates and single point energy of **3** after geometry optimisation

1.C	-1.960074	-2.136577	-4.340501
2.C	-4.540581	-0.664137	-3.715816
3.C	4.474099	-1.399447	-3.594563
4.C	0.196364	3.806789	-3.568474
5.C	-1.889531	0.640695	-2.955951
6.C	1.524878	-1.553266	-2.827147
7.C	3.323000	2.583401	-2.585890
8.C	4.511059	2.994799	-1.712728
9.C	1.791082	5.410967	-1.544898
10.C	3.611323	-3.449965	-1.529887
11.C	-3.676992	-2.981045	-1.412138
12.C	5.530357	0.874576	-0.952711
13.C	-4.981862	-2.639173	-0.688217
14.C	-0.366311	3.508041	-0.530708
15.C	4.908359	-0.407367	-0.392892
16.C	-4.906997	0.407946	0.394237
17.C	0.365998	-3.508882	0.527611
18.C	4.980118	2.639319	0.688391
19.C	-5.530208	-0.873724	0.953219
20.C	3.675241	2.981313	1.412115
21.C	-1.791412	-5.411823	1.543308
22.C	-3.608911	3.448901	1.530565
23.C	-4.511888	-2.994719	1.712813
24.C	-3.322820	-2.584615	2.585483

25.C	-1.523089	1.552330	2.829816
26.C	1.888420	-0.639100	2.957977
27.C	-4.472377	1.397609	3.595424
28.C	-0.195077	-3.805069	3.565878
29.C	4.539775	0.668421	3.717419
30.C	1.959251	2.138968	4.340207
31.H	-1.957289	-1.655209	-5.332354
32.H	-4.475901	-0.230020	-4.727170
33.H	-2.494234	-3.094416	-4.443799
34.H	4.323515	-2.180796	-4.357730
35.H	0.958710	3.969244	-4.346793
36.H	-0.918751	-2.358182	-4.066788
37.H	4.305303	-0.426775	-4.082839
38.H	-5.154770	-1.574845	-3.796714
39.H	-1.923788	1.134493	-3.940142
40.H	-0.344568	2.881682	-3.813234
41.H	-0.516865	4.645330	-3.629478
42.H	1.407943	-2.255453	-3.668198
43.H	5.531563	-1.448224	-3.290470
44.H	3.171173	3.348357	-3.369974
45.H	-5.082540	0.056253	-3.083405
46.H	3.564162	1.649901	-3.122017
47.H	1.321202	-0.541261	-3.207871
48.H	-0.835509	0.530032	-2.668688
49.H	3.476013	-4.176575	-2.348042
50.H	5.448706	3.083098	-2.293580
51.H	-3.916518	-3.384585	-2.413343
52.H	2.603648	5.622727	-2.257684
53.H	-2.370964	1.312778	-2.229882
54.H	5.574785	0.788494	-2.045982
55.H	0.760685	-1.793465	-2.076317
56.H	1.040872	6.209502	-1.667502
57.H	4.293264	3.976990	-1.273094
58.H	-5.530502	-1.905153	-1.292710
59.H	4.643600	-3.560801	-1.162142
60.H	-3.160166	-3.795634	-0.877238
61.H	2.929864	-3.726760	-0.713028
62.H	6.560960	1.037273	-0.582657
63.H	5.525075	-1.271074	-0.702439
64.H	-5.632620	-3.524399	-0.555826
65.H	2.200733	5.498949	-0.526554
66.H	-4.950490	0.389162	-0.708177
67.H	-0.956602	2.593509	-0.675247
68.H	-1.054492	4.367304	-0.573301
69.H	-0.066371	-3.478344	-0.483703
70.H	5.631612	3.523965	0.556915
71.H	-6.560908	-1.035002	0.584272
72.H	0.065756	3.477494	0.480681
73.H	-2.201031	-5.499529	0.524868
74.H	1.054242	-4.368073	0.569798
75.H	-5.522750	1.272220	0.703604
76.H	4.951860	-0.388786	0.709032
77.H	0.956285	-2.594358	0.671850
78.H	-2.926812	3.725609	0.713931
79.H	3.158512	3.795783	0.875940

80.H	-4.640910	3.559184	1.161579
81.H	5.528226	1.904827	1.293898
82.H	-4.295446	-3.977182	1.273502
83.H	-1.042106	-6.211151	1.666214
84.H	-5.573951	-0.787829	2.047325
85.H	-2.604358	-5.622276	2.256026
86.H	-5.449210	-3.082254	2.295009
87.H	-3.474669	4.175910	2.348312
88.H	3.913630	3.385400	2.412755
89.H	-0.757847	1.792874	2.079728
90.H	2.368982	-1.312138	2.232030
91.H	0.834282	-0.527543	2.670263
92.H	5.085179	-0.048694	3.084499
93.H	-5.529574	1.441216	3.289813
94.H	-3.562643	-1.650885	3.121583
95.H	-1.319658	0.540169	3.210244
96.H	-3.170612	-3.349598	3.368944
97.H	0.518992	-4.642699	3.628231
98.H	5.150553	1.581036	3.801502
99.H	-1.406941	2.253840	3.671171
100.H	0.344816	-2.878888	3.808636
101.H	1.921828	-1.132077	3.942255
102.H	-4.299849	0.426800	4.085546
103.H	-4.326028	2.181092	4.356999
104.H	-0.957261	-3.966749	4.344468
105.H	0.917847	2.361079	4.066624
106.H	2.493769	3.096750	4.442729
107.H	4.474122	0.232234	4.727616
108.H	1.956485	1.658348	5.332188
109.N	2.133195	2.402458	-1.730283
110.N	-2.827766	-1.774761	-1.473623
111.N	3.510943	-0.509683	-0.859439
112.N	4.654370	2.016326	-0.612212
113.N	0.541388	0.234402	-0.071633
114.N	-0.541471	-0.235161	0.072424
115.N	-4.655362	-2.016343	0.613221
116.N	-3.509456	0.509251	0.860268
117.N	2.825855	1.774984	1.473163
118.N	-2.134132	-2.404404	1.728191
119.Si	-2.785094	-1.009893	-3.058333
120.Si	3.271740	-1.688354	-2.144095
121.Si	0.968442	3.716739	-1.839155
122.Si	-0.967844	-3.718150	1.836690
123.Si	-3.269256	1.687864	2.145187
124.Si	2.783830	1.011408	3.058469
125.Ti	2.378368	1.029950	-0.313447
126.Ti	-2.378933	-1.030592	0.313623

Energy: –684.70311614 eV

Table S2. Final coordinates and single point energy of **4** after geometry optimisation

1.C	-3.338243	-1.103299	-4.621161
2.C	2.886283	0.979193	-4.267659
3.C	-3.944305	1.808317	-3.992519

4.C	4.820656	-1.303042	-3.655776
5.C	-1.092340	0.730601	-3.598903
6.C	1.889203	-1.621916	-2.924359
7.C	-1.226847	-3.720294	-2.208105
8.C	-4.797534	-0.250256	-1.581136
9.C	-3.946481	-4.626627	-1.223124
10.C	4.872974	1.000998	-1.273760
11.C	1.819465	3.593081	-1.258665
12.C	-1.975033	3.329333	-0.670562
13.C	-5.204768	-1.277047	-0.524789
14.C	5.690061	0.438404	-0.115952
15.C	-4.891758	3.331884	0.183280
16.C	-1.515839	-5.460938	0.316593
17.C	4.024977	4.825689	0.475716
18.C	1.199613	4.880898	1.490732
19.C	-5.060726	0.071796	1.541812
20.C	4.145245	-1.826880	1.514884
21.C	-4.338023	-2.284113	1.575405
22.C	-2.844499	-2.638347	1.663742
23.C	4.988073	1.749451	1.860037
24.C	5.095347	-0.716690	1.966708
25.C	-2.824380	4.054531	2.217003
26.C	-3.907484	0.793386	2.236683
27.C	3.604575	2.165762	2.375793
28.C	1.600411	-3.772607	2.973513
29.C	0.297791	-1.005639	3.279854
30.C	2.953250	-1.636341	4.619654
31.H	-3.108043	-0.809184	-5.658335
32.H	2.761524	0.550170	-5.275622
33.H	-3.693529	2.058169	-5.036540
34.H	-4.405696	-1.374143	-4.590858
35.H	-0.921589	1.227100	-4.567644
36.H	4.580118	-1.751136	-4.634213
37.H	3.690560	1.730205	-4.326753
38.H	-2.757416	-2.010090	-4.394367
39.H	-5.026730	1.608020	-3.957190
40.H	5.700046	-0.658388	-3.808310
41.H	1.955774	1.503908	-4.004154
42.H	1.598151	-1.951462	-3.933312
43.H	-3.753406	2.705956	-3.384180
44.H	-0.465340	-0.169966	-3.572925
45.H	-1.678571	-4.298405	-3.028256
46.H	5.117141	-2.121117	-2.980021
47.H	-0.742573	1.394840	-2.797543
48.H	-5.308724	-0.557874	-2.512737
49.H	2.208567	-2.520518	-2.369507
50.H	-1.208836	-2.668538	-2.527666
51.H	5.488848	0.934206	-2.189427
52.H	1.007615	-1.172578	-2.444980
53.H	-4.416150	-3.969307	-1.970397
54.H	-0.187236	-4.078732	-2.131662
55.H	-3.782755	-5.602891	-1.710200
56.H	2.639568	3.252819	-1.908803
57.H	1.442711	4.548695	-1.653567
58.H	-2.349478	2.889222	-1.604447

59.H	-5.236148	0.732097	-1.321703
60.H	1.022394	2.841207	-1.348611
61.H	4.710191	2.082814	-1.109201
62.H	-4.916263	-2.271600	-0.877767
63.H	-5.198350	2.779718	-0.717412
64.H	-1.946184	4.422604	-0.798934
65.H	-4.667637	-4.791227	-0.407577
66.H	-6.305311	-1.266993	-0.388641
67.H	-1.482926	-6.341993	-0.344867
68.H	5.925698	-0.610661	-0.342368
69.H	-0.940148	2.976762	-0.571820
70.H	4.799726	4.377728	-0.165195
71.H	-4.918901	4.406707	-0.062848
72.H	6.647155	0.979423	0.021768
73.H	3.792378	5.820154	0.060787
74.H	-0.489056	-5.291289	0.682008
75.H	4.413149	-2.134436	0.488872
76.H	-5.648868	3.162031	0.965316
77.H	-5.542633	0.771748	0.847441
78.H	-2.122991	-5.742926	1.191258
79.H	1.239738	5.917224	1.116716
80.H	-4.897374	-3.108348	1.109235
81.H	5.343332	2.521220	1.165525
82.H	4.460537	4.986936	1.474561
83.H	0.144395	4.574387	1.403846
84.H	-2.968229	5.097683	1.891242
85.H	6.155963	-1.037814	1.939066
86.H	-2.737188	-3.638979	2.125719
87.H	4.324539	-2.719597	2.146818
88.H	-5.830612	-0.284151	2.254978
89.H	0.800296	-4.061217	2.271238
90.H	1.455068	4.916319	2.562850
91.H	-2.376475	-1.945829	2.391741
92.H	-4.764663	-2.129640	2.580754
93.H	5.729230	1.671135	2.679923
94.H	-1.816668	4.002841	2.661827
95.H	-0.284195	-0.888485	2.352603
96.H	2.501364	-4.344014	2.697553
97.H	-4.317266	1.645249	2.813128
98.H	-3.540584	3.872954	3.034376
99.H	3.703499	3.132829	2.905140
100.H	4.853999	-0.453073	3.005282
101.H	-3.468506	0.120689	2.999107
102.H	3.283545	1.443296	3.148568
103.H	1.281576	-4.131733	3.965843
104.H	0.474507	-0.014463	3.726985
105.H	-0.325551	-1.563659	3.994497
106.H	3.910587	-2.180761	4.611648
107.H	3.172064	-0.573662	4.808638
108.H	2.375455	-2.014624	5.479486
109.K	0.602372	-2.434617	-0.065744
110.K	-0.196586	1.810665	1.789877
111.N	-3.329815	-0.138902	-1.768223
112.N	3.589912	0.275995	-1.393662
113.N	-0.538867	0.007197	-0.270856

114.N	0.745071	0.207088	-0.121924
115.N	-2.181604	-2.524238	0.350926
116.N	-4.502268	-1.051546	0.758877
117.N	4.876959	0.472616	1.119975
118.N	-2.888682	1.199730	1.240495
119.N	2.621519	2.211917	1.270170
120.N	2.739016	-1.348483	1.558720
121.Si	-2.916284	0.310455	-3.416299
122.Si	3.297791	-0.363198	-2.992930
123.Si	-2.256192	-3.968931	-0.626392
124.Si	2.430322	3.784039	0.515212
125.Si	-3.135438	2.863067	0.746071
126.Si	1.956275	-1.893380	3.017853
127.Ti	-2.321057	-0.398261	-0.066920
128.Ti	2.517873	0.360079	0.320473

Energy: −688.35399449 eV

Table S3. Final coordinates and single point energy of **5** after geometry optimisation

1.C	2.373883	-1.541036	-5.234536
2.C	4.752852	0.742205	-2.967808
3.C	-2.284134	0.847812	-2.863395
4.C	-5.186815	-0.063456	-2.991567
5.C	2.571827	3.021112	-2.929252
6.C	0.134866	-1.812459	-3.188578
7.C	4.251820	-0.649401	-2.581011
8.C	2.683076	-3.609207	-3.073503
9.C	-0.441395	4.506783	-2.936300
10.C	4.024795	3.030248	-2.454405
11.C	-4.369226	2.029304	-0.975376
12.C	5.273883	1.743669	-0.775189
13.C	-4.447784	-1.925291	-0.503438
14.C	1.480451	5.424137	-0.782777
15.C	0.668381	-3.367190	0.085163
16.C	-1.473854	-5.423813	0.774531
17.C	-0.663125	3.370426	-0.085380
18.C	4.445400	1.927377	0.502391
19.C	-5.275357	-1.745106	0.775144
20.C	4.377746	-2.023129	0.978486
21.C	-4.024151	-3.032627	2.452095
22.C	0.442209	-4.506499	2.934628
23.C	-2.680793	3.609324	3.074580
24.C	-4.252528	0.645843	2.587373
25.C	5.182555	0.072214	2.995985
26.C	-2.570959	-3.023731	2.925909
27.C	-0.134849	1.810449	3.187549
28.C	-4.755548	-0.746446	2.969158
29.C	2.280993	-0.849735	2.858374
30.C	-2.370137	1.542658	5.238671
31.H	1.900887	-2.328488	-5.847860
32.H	1.968198	-0.570005	-5.561340
33.H	3.452775	-1.551376	-5.463960
34.H	4.262015	1.034788	-3.907405
35.H	-2.092182	0.042035	-3.587270

36.H	-5.305786	0.749754	-3.728181
37.H	2.488116	2.345276	-3.803795
38.H	5.854977	0.746313	-3.143159
39.H	-2.479912	1.774920	-3.426904
40.H	4.582340	-1.345969	-3.380042
41.H	-4.972824	-0.989487	-3.550679
42.H	4.724327	3.345527	-3.264975
43.H	0.214545	4.880753	-3.740110
44.H	-0.291274	-1.060189	-3.868043
45.H	2.166263	-4.342950	-3.716591
46.H	2.352517	4.034597	-3.326596
47.H	-0.304424	-2.789251	-3.450673
48.H	-1.064320	3.702800	-3.357712
49.H	3.760954	-3.668320	-3.301710
50.H	-1.365509	0.981494	-2.273828
51.H	-6.158732	-0.191168	-2.485692
52.H	-1.107806	5.335161	-2.639372
53.H	4.793794	-0.976849	-1.672562
54.H	4.110370	3.757399	-1.634284
55.H	-4.479227	2.831072	-1.726208
56.H	-0.177848	-1.541786	-2.170486
57.H	2.544071	-3.915474	-2.026219
58.H	-5.150858	-1.982287	-1.356959
59.H	6.045761	2.541111	-0.891042
60.H	2.200230	5.820804	-1.519495
61.H	0.194171	-3.403007	-0.904732
62.H	5.797568	0.778581	-0.712295
63.H	-2.030741	-5.178126	-0.144596
64.H	-3.960908	-2.919614	-0.458061
65.H	1.520885	-4.065206	0.077224
66.H	-5.345668	1.891442	-0.480783
67.H	-3.654567	2.371957	-0.211628
68.H	0.773409	6.237347	-0.540416
69.H	-0.765747	-6.236162	0.532661
70.H	-1.044081	2.348349	-0.218162
71.H	1.047218	-2.344367	0.218436
72.H	5.354986	-1.881636	0.486409
73.H	2.039944	5.180428	0.135230
74.H	3.959585	2.922158	0.454253
75.H	3.666270	-2.369314	0.213354
76.H	-1.514135	4.069970	-0.076048
77.H	-5.800541	-0.780669	0.714158
78.H	-6.045763	-2.543983	0.890530
79.H	-2.195883	-5.821905	1.508186
80.H	-0.186941	3.405398	0.903380
81.H	5.148117	1.986021	1.356284
82.H	-2.537772	3.915509	2.027634
83.H	4.489272	-2.823724	1.730515
84.H	-4.110371	-3.759006	1.631253
85.H	-4.792784	0.976547	1.678769
86.H	1.107457	-5.336204	2.638670
87.H	0.176236	1.544596	2.167637
88.H	6.156684	0.192759	2.492700
89.H	-3.759054	3.670648	3.299878
90.H	1.363918	-0.981551	2.265936

91.H	1.066150	-3.703210	3.355642
92.H	-2.164189	4.342171	3.719074
93.H	-2.351315	-4.037316	3.322603
94.H	-0.214705	-4.878661	3.738385
95.H	-4.722930	-3.348776	3.262998
96.H	-5.858096	-0.750732	3.141199
97.H	4.969384	1.003267	3.546892
98.H	0.305546	2.785690	3.453812
99.H	-4.583618	1.340787	3.387896
100.H	5.295854	-0.735730	3.739183
101.H	0.291979	1.054442	3.862419
102.H	2.475723	-1.778708	3.419006
103.H	-2.487078	-2.348064	3.800526
104.H	-4.267316	-1.041496	3.909267
105.H	2.086622	-0.046878	3.584899
106.H	-3.448873	1.550006	5.468614
107.H	-1.899020	2.331623	5.851428
108.H	-1.961873	0.572745	5.565741
109.N	2.796130	-0.659293	-2.365598
110.N	4.374084	1.707180	-1.933005
111.N	1.663496	2.605500	-1.850922
112.N	-3.444402	-0.870102	-0.647170
113.N	0.551389	0.176338	-0.269919
114.N	-0.556580	-0.178576	0.264990
115.N	3.440449	0.873785	0.648886
116.N	-1.663234	-2.607414	1.847349
117.N	-4.374548	-1.708874	1.932726
118.N	-2.796734	0.653835	2.375081
119.Si	2.008774	-1.836102	-3.363646
120.Si	-3.762739	0.402522	-1.777559
121.Si	0.552286	3.861735	-1.425371
122.Si	-0.549306	-3.861348	1.422152
123.Si	3.762369	-0.398289	1.778876
124.Si	-2.008337	1.834984	3.366666
125.Ti	2.117012	0.760433	-0.978314
126.Ti	-2.120551	-0.761965	0.977938

Energy: –685.31888924 eV

References

1. C.C Cummins, R.R Schrock, W.D Davis, *Organometallics* **1992**, *11*, 1452.
2. C.C Cummins, J. Lee, R.R Schrock, W.D Davis, *Angew. Chem. Int. Ed. Engl.* **1992**, *31*, 1501.
3. I. S. Weitz, M. Rabinovitz, *Chem. Soc., Perkin Trans.* **1993**, *1*, 117.
4. T. A. Scott, B. A. Ooro, D. J. Collins, M. Shatruk, A. Yakovenko, K. R. Dunbarc, H-C Zhou, *Chem. Commun.* **2009**, 65.
5. M. Brookhart, B. Grant, A. F. Volpe Jr, *Organometallics* **1992**, *11*, 3920.
6. P. J. Hill, L. R. Doyle, A. D. Crawford, W. K. Myers, A. E. Ashley, *J. Am. Chem. Soc.* **2016**, *138*, 13521.
7. M. W. Weatherburn, *Anal. Chem.* **1967**, *39*, 971.
8. G. W. Watt, J. D. Chrisp, *Anal. Chem.* **1952**, *24*, 2006.
9. C. Fonseca Guerra, J. G. Snijders, G. te Velde, E. J. Baerends, *Theor. Chem. Acc.* **1998**, *99*, 391.
10. G. te Velde, F. M. Bickelhaupt, S. J. A. van Gisbergen, C. Fonseca Guerra, E. J. Baerends, J. G. Snijders T. Ziegler, *J. Comput. Chem.* **2001**, *22*, 931.
11. S. H. Vosko, L. Wilk, M. Nusair, *Can. J. Phys.* **1980**, *58*, 1200.
12. A. D. Becke, *Phys. Rev. A.* **1988**, *38*, 3098.
13. J. P. Perdew, *Phys. Rev. B.* **1986**, *33*, 8822.
14. S. Portmann, H. P. Luthi, *Chimia* **2000**, *54*, 766.
